# OECD-407 Driven 28-day-repeated-dose non-clinical safety evaluation of *Tinospora cordifolia* (Giloy) stem aqueous extract in Sprague-Dawley rats under GLP compliance

**DOI:** 10.3389/fphar.2023.1095083

**Published:** 2023-05-18

**Authors:** Acharya Balkrishna, Swati Haldar, Anurag Varshney

**Affiliations:** ^1^ Drug Discovery and Development Division, Patanjali Research Institute, Governed by Patanjali Research Foundation Trust, Haridwar, Uttarakhand, India; ^2^ Department of Allied and Applied Sciences, University of Patanjali, Haridwar, Uttarakhand, India; ^3^ Patanjali UK Trust, Glasgow, United Kingdom; ^4^ Special Centre for Systems Medicine, Jawaharlal Nehru University, New Delhi, India

**Keywords:** 28-day repeated dose toxicity, 14-day recovery period, *Tinospora cordifolia* (Wild.) Hook.f. & Thomson (Giloy), OECD 407, GLP, ayurveda

## Abstract

**Introduction:** *Tinospora cordifolia* (Wild.) Hook.f. & Thomson (Giloy), has been widely used in the Ayurvedic system of medicine. However, some sporadic under-powered case studies have recently reported *Tinospora cordifolia* associated toxicity. Thus, following OECD 407 guidelines, a 28-day-repeated-dose-14-day-recovery toxicological evaluation of the aqueous extract of *T. cordifolia* stem (TCWE) was conducted under good laboratory practice (GLP), in Sprague-Dawley (SD) rats.

**Methods:** 100, 300, and 1000 mg/kg/day of TCWE was given orally to designated treatment groups of either sex. Two separate 14-day recovery satellite groups received either vehicle control or 1000 mg/kg/day of TCWE.

**Results:** In this study, TCWE was found safe up to a dose of 1000 mg/kg/day with no mortality or related toxicological manifestation in terms of clinical signs, ocular effects, hematology, urinalysis, clinical chemistry parameters, or macro- or microscopic changes in any organs. The satellite group did not show any adverse effect after 14-day recovery period. Thus, the No-Observed-Adverse-Effect-Level (NOAEL) of TCWE was determined to be 1000 mg/kg/day.

**Discussion:** In conclusion, this study established the non-clinical safety of the aqueous extract of *T. cordifolia* stem, which confirms the age-old safe medicinal use of this herb, and also paves the path for future clinical research on formulations containing *Tinospora cordifolia*.

## 1 Introduction

*Tinospora cordifolia* is a member of Menispermaceae family which is indigenous to the tropical habitats of the Indian sub-continent. Locally known as Giloy or Guduchi, *Tinospora cordifolia* grows as a climber with heart-shaped leaves (Upadhyay et al., 2010). Members of Menispermaceae family are known for being rich sources of phytocompounds, like, alkaloids, polysaccharides and terpenes, with reported anti-oxidant (Imtiyaj Khan et al., 2011), anti-inflammatory (Tiwari et al., 2014), anti-diabetic (Sharma et al., 2013), immune-stimulatory (Sharma et al., 2012a) activities, among several other medicinal properties (Kapil and Sharma, 1997; Cho et al., 2001). *T. cordifolia* is a popular medicinal herb used in different Ayurvedic formulations. An earlier computational study from our group showed that tinocordiside, one of the major phytocompounds present in *T. cordifolia* could potentially inhibit host-pathogen interaction during COVID-19 infection by disrupting electrostatic interactions between human host ACE2 receptor and SARS-CoV-2 viral spike (S) protein (Balkrishna et al., 2021b). Giloy Ghanvati, the aqueous extract of *T. cordifolia* in the form of tablets, effectively attenuated SARS-CoV-2 viral recombinant spike (S) protein-induced pathological symptoms in humanized zebrafish, which had their swim bladders xenotransplanted with human A549 lung cells (Balkrishna et al., 2021a).

*T. cordifolia*, in different forms such as extract, decoction and pills, almost became a household medicine in India to fight against COVID-19. Pilot-scale clinical trial (CTRI/2020/05/025273) conducted by Devpura et al. (2021) (Devpura et al., 2021) has also included *T. cordifolia* as one of the components administered orally to the COVID-19 patients. Otherwise also, different parts of *T. cordifolia*, namely, roots, stems and leaves, have an array of reported medicinal uses, such as, metabolism enhancement, endocrine modulation, anti-oxidative, anti-toxic, anti-biotic, anti-parasitic, anti-cancer, anti-hyperlipidemic, anti-anxiety, anti-arthritic, cardio-protective, hepato-protective, nephro-protective, neuro-protective, osteo-protective and adaptogenic (Saha and Ghosh, 2012; Dhama et al., 2017). In fact, there are more than 25 patents describing the medicinal importance of this plant (Dhama et al., 2017). G1-4A, a known polyclonal B cell mitogen from the crude extract of dried *T. cordifolia* stems, has been reported to enhance immune response in mice, through macrophage activation and IL-1 secretion (Raghu et al., 2009). The stem extracts of *T. cordifolia* are known for their ability to regulate blood glucose (Sangeetha et al., 2011). Jatrorrhizine, magnoflorine and palmatine rich fraction from *T. cordifolia* stem could impart insulin mimicry and trigger insulin releasing both under *in vitro* and *in vivo* conditions (Patel and Mishra, 2011). The crude extract of *T. cordifolia* stems in different solvents, like, hexane, chloroform, dichloromethane and ethyl acetate could inhibit the enzymatic activities of salivary and pancreatic amylases, and glucosidase (Chougale et al., 2009), thus, implying therapeutic potential against diabetes mellitus (Saha and Ghosh, 2012). Irrespective of its prominence in Ayurveda, a few recent case reports have linked apparent hepatotoxicity in clinical subjects with consumption of *T. cordifolia* (Nagral et al., 2021; Kulkarni et al., 2022). From safety point of views, a single study has reported non-toxicity of *T. cordifolia* at a single dose of 2000 mg/kg/day (Ghatpande et al., 2019). However, to the best of our knowledge there is no report on repeated dose toxicity assessments of *T. cordifolia*. Therefore, a 28-day repeated dose with 14-day recovery study as per OECD 407 guidelines was conducted, which is now reported in this manuscript. Classically water has been the choice of solvent for the extraction of medicinal herbs, although water and alcohols (ethanol, and methanol at times) have been extensively used nowadays with regulatory compliance. Since our study is aimed in the line of classical preparation, we used water as the solvent to extract *T. cordifolia* stems. This is also in line with green extraction process with superior safety modules. Different doses of the aqueous extract prepared from the stem of *Tinospora cordifolia* (Wild.) Hook.f. & Thomson (TCWE) was administered orally to Sprague-Dawley rats of either sex for consecutive 28 days. A separate group of study animals, which were given highest dose of TCWE was allowed to recover for subsequent 14 days before conducting requisite toxicological evaluations.

Currently, the regulatory requirements for establishing safety and efficacy of herbal and/or traditional medicines are not as well-defined as those for modern drugs. This infuses an undercurrent of limited clinical acceptability of these traditional/herbal medicines, despite, they being in use for several centuries without side-effects. *T. cordifolia* is used as an ingredient in many traditional and herbal medicines. A GLP regulatory toxicological study establishing the non-clinical safety of *T. cordifolia*, such as the one reported here, would pave the path for subsequent clinical studies as per the regulatory requirements for drug development. This in turn would improve the clinical acceptability of the medicines containing this herb. In addition, this study is likely to expand the scope of developing *T. cordifolia* alone, in combination or as a source of phytopharmaceutical/s in modern medicine.

## 2 Materials and methods

### 2.1 Chemicals, reagents and test item

The whole plant of *Tinospora cordifolia* (Wild.) Hook.f. & Thomson was obtained from the Patanjali Research Institute’s herbal garden in Haridwar, India. The mature whole plant collection was done in accordance with local, regional, and global regulations, and its authenticity certified by the Council of Scientific and Industrial Research-National Institute of Science Communication and Information Resources (CSIR-NISCAIR), New Delhi, Government of India (vide authentication Voucher No. NISCAIR/RHMD/Consult/2019/3453-54-63) and the Patanjali Research Foundation Herbarium (through Collection No. 6436 and Accession No. 12215), in the month of May, 2021 for preparation of the aqueous extract used in the current study. The aqueous extract from the stem of the authenticated *T. cordifolia* whole plant (TCWE) (batch number D4/CHM/SOLE092/0521; Manufacture date: May 2021; Expiry date: April 2024) in the form of brown color powder was prepared at Patanjali Research Institute. An aqueous suspension of TCWE was prepared for oral administration to the study animals. The best commercial quality chemicals and reagents were used for the rest of the experiments.

### 2.2 Preparation of TCWE

*T. cordifolia* extract was prepared as per an in-house optimized protocol, following the recommendations in Ayurvedic pharmacopeia of India. Approximately 30 kg of dried *T. cordifolia* whole plant material was extracted using 210 L of water as the solvent at 75°C–80°C for 4 h in an extractor under reflux conditions. The extraction process was repeated twice under same conditions. The extract was filtered using a 10 μ filter cloth. The filtrate was pooled, concentrated, and spray dried at an intake air temperature of 140°C and a feed flow rate of 7 L/hr to produce a light to dark brown tinted powder with a 19% dry yield.

### 2.3 Compositional analysis of TCWE

Phytochemical composition of TCWE was analysed through Ultra High Performance Liquid Chromatography (UHPLC), as per OECD Q2R1 guidelines. TCWE was dissolved in hydro-methanol at a concentration of 50 mg/mL by sonication for 30 min, and subsequently filtered through 0.45 μ nylon filter before UHPLC analysis. Based on an earlier report, magnoflorine and β-ecdysone were chosen as the marker phytocompounds of *T. cordifolia* aqueous extract (Sharma et al., 2019). Therefore, stock solutions (1 mg/mL) of magnoflorine (Chem faces, Hubei, Batch. No. CFS202101) and β-ecdysone (PHY-proof, Germany, Batch. No. 77100776) standards were prepared by dissolving precisely weighed amounts in methanol. 10 μL of standards and test solution were injected. Analysis was performed using the Nexera XR UHPLC system (Shimadzu, Japan). Separation was achieved on a Shimdzu-C18 (5 μm, 4.6 × 250 mm) column through gradient elution using solvent A (0.1% orthophosphoric acid in water, pH adjusted to 2.5 with diethylamine) and solvent B [0.1% orthophosphoric acid in acetonitrile: water (88:12), pH adjusted to 2.5 with diethylamine]. The gradient program used for elution allowed a flux of steadily varying proportions of solvents A and B in the mobile phase as mentioned here: 90:10 for 0–10 min, 85:15 from 10–20 min, 75:25 from 20–30 min, 65:35 from 30–40 min, 60:40 from 40–45 min, 40:60 from 45–55 min, 10:90 from 55–60 min and back to 90:10 from 60–70 min. A steady flow rate of 1.0 mL/min was maintained throughout the process. Detection of the phytocompounds were done at 254 nm.

### 2.4 Study facility and ethical declaration

The current 28-day repeated dose 14-day recovery non-clinical safety assessment of TCWE was conducted in full compliance with the Organization of Economic Co-operation and Development (OECD) Principles of Good Laboratory Practice (GLP) for the Testing of Chemicals as specified by International Legislation [OECD ENV/MC/CHEM (98)17] at Vanta Bioscience Limited, Gummidipundi, Tamil Nadu, India. The test facility is certified by National Good Laboratory Practice (GLP) Compliance Monitoring Authority (NGCMA), Department of Science & Technology, Government of India (GLP/C-175/2021 dated 29 October 2021; validity: 18 July 2021—17 July 2024); and by the Committee for the Purpose of Control and Supervision of Experiments on Animals (CPCSEA) for ‘Research for commercial purpose and In-house breeding on small animals’ (Reg. No. 1784/PO/RcBiBt/S/2014/CPCSEA dated 28 August 2017), Department of Animal Husbandry and Dairying, Ministry of Fisheries, Animal Husbandry and Dairying, Government of India.

The experimental procedures and animal husbandry practices followed in the current study conformed to the CPCSEA standards (CPCSEA, 2018), and were prior approved by the Institutional Animal Ethics Committee (IAEC) of Vanta Bioscience Limited, Gummidipundi, Tamil Nadu, India (vide IAEC Protocol No. 31/21). The study was conducted in compliance with the ARRIVE guidelines (du Sert et al., 2020).

TCWE was provided as coded test article (CHOS/GIWA/1021/394) to Vanta Bioscience Limited by Patanjali Research Institute, Haridwar, India. Therefore, the experimenters who performed the current non-clinical safety assessment were blinded to the identity of the test article, TCWE. The identity of TCWE was disclosed on submission of the final report. Microbial load of TCWE was conducted in-house at Patanjali Research Institute, and the test article was provided to Vanta Bioscience Limited only after all these parameters were clean as per the guidelines of Ayurvedic Pharmacopoeia of India (API). TCWE was free of specific pathogens and also did not have any aerobic bacterial and yeast and mold contaminations. Loss on drying (LoD) of the extract was observed to 4.51%. The heavy metal, such as, Lead, Arsenic, Mercury and Cadmium contents were found to be within permissible limits. Aflatoxin and pesticide levels were also below limits of quantification (BLQ).

### 2.5 Non-clinical safety assessment of TCWE

**FIGURE 1:**
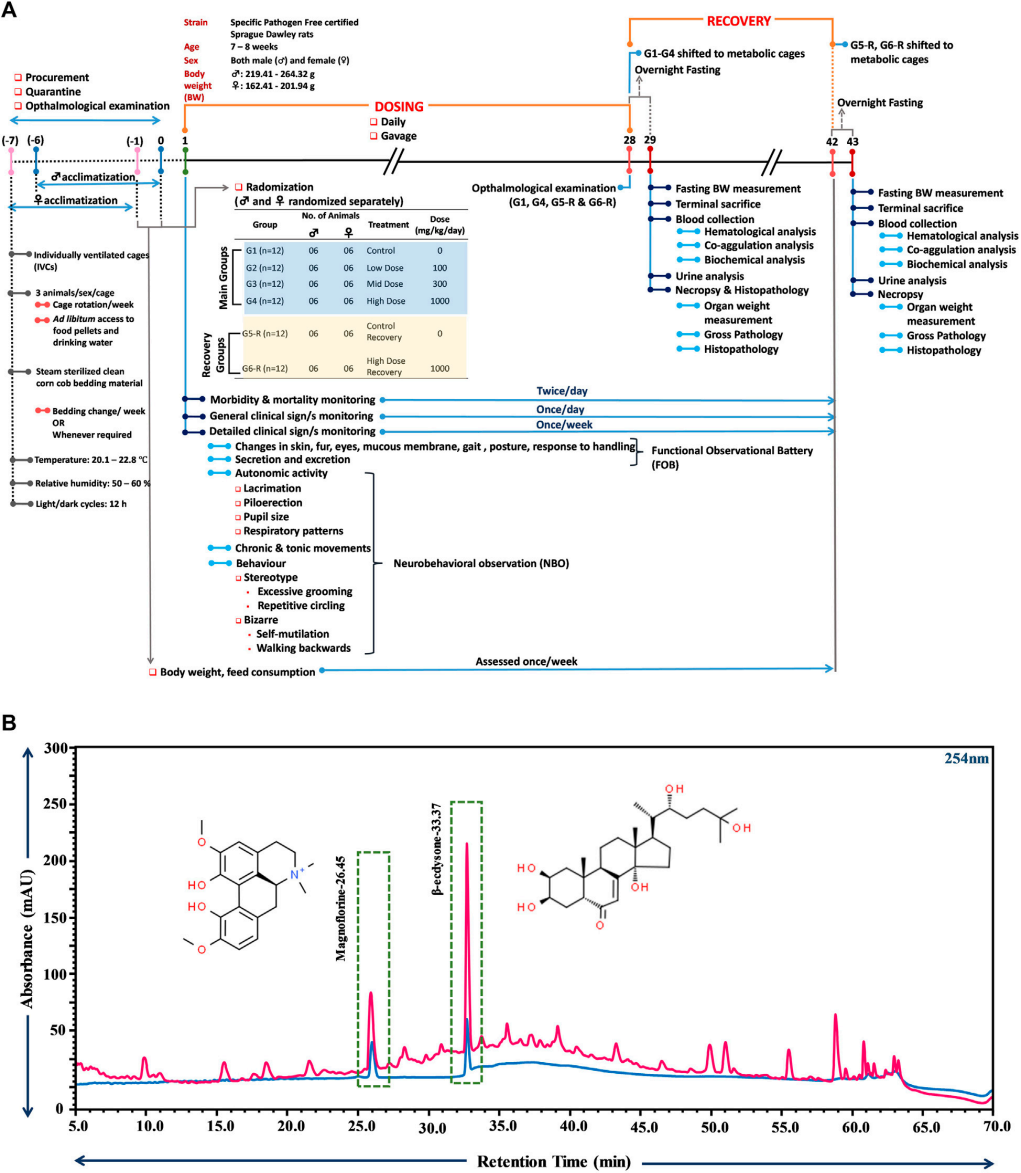
Schematic illustration of the study plan and compositional analysis of TCWE through UHPLC. **(A)** The schematic provides a comprehensive detail of the experiment carried out according to the OECD 407 guidelines, depicting the various phases of the experiment, namely, dosing (1–28 days) and recovery (29–43 days). Start of dosing is taken as day 1. One week prior to that, animals were procured, acclimatized and subjected to ophthalmological examination. The housing conditions are also mentioned along with randomization and grouping information. Information on the timings and frequencies of different experimental read-outs, such as, body weight and feed consumption, morbidity and mortality, clinical signs, blood collection (for haematological and clinical chemistry analyses), urine analysis, and necropsy and histopathology is also provided. **(B)** Overlap chromatograms of 20 ppm standard mix of phytocompounds (blue) and TCWE (pink) showing the presence of magnoflorine and β-ecdysone with retention times of 26.45 and 33.37 min, respectively.

A comprehensive schematic of the entire study mentioning all the experimental details is provided in Figure 1A. The entire experiment was of 50-day duration, with randomization completion marked as day 0 in the timeline shown in Figure 1A. Procurement, quarantine and ophthalmological examination were conducted prior to randomization. Acclimatization of the study animals also overlapped with this pre-randomization period. Daily dosing of TCWE started on day 1 and continued till day 28 in all groups. On day 29, study animals belonging to the main groups (G1-G4), after overnight fasting, were terminally sacrificed and subjected to end-point analyses. The animals belonging to the satellite groups (G5-R, G6-R) were put on a 14-day dosing free recovery till day 42. These animals were also terminally sacrificed on day 43, after overnight fasting, and subjected to end-point analyses.

#### 2.5.1 Study animal procurement, acclimatization and husbandry

Specific Pathogen Free (SPF), 6–7 weeks-old, male (weighing 219.41–264.32 g) and female (weighing 162.41–201.94 g) Sprague-Dawley (SD) rats, were procured from CPCSEA approved vendor, Hylasco Biotechnology (India) Pvt. Ltd., Telangana, India. Prior to inclusion in the study, animals were examined by the study veterinarian for their health status and suitability for use in the experiment. Animals were acclimatized to the test conditions prior to administration of the test article, for 5 days in case of males and for 6 days in case of females. The animals of same sex were housed three per individually ventilated cage(s) (IVC) of 41 cm × 41 cm × 78 cm dimension (Optirat^®^ Plus, Animal Care Systems, Inc., United States), with facilities for holding pelleted food (Lot No.: 195 from Krishna Valley Agrotech LLP, Date of Manufacture: 03.09.2021 and Expiry Date: 02.03.2022) and drinking water [reverse osmosis (RO) purified] *ad libitum*. The study animals had *ad libitum* access to feed and water throughout the experiment, except during overnight fastings on days 28 (for main groups) and 43 (for recovery groups). Steam sterilized bedding material (Corn Cob, Matha Agrotech, Batch No: CCG/MA-05/June/2021, Manufacturing Date: June 2021, Best Before: June 2022) were provided in each cage and changed once a week or whenever required. Cage rotation was carried out once a week. The environment was maintained at 20.1°C–22.8°C with 50–63% relative humidity and adequate fresh air supply (minimum 12 air changes/hr). Light and dark cycles of 12 h each were maintained throughout the experimental phase.

#### 2.5.2 Randomization, formulation preparation and dosing

Subsequent to acclimatization and thorough medical check-up, a total of 72 SD rats (36 males and 36 females) were randomly allocated to six different dose groups (4 main groups and 2 recovery groups). Each main group (G1, G2, G3 and G4) and recovery group (G5-R and G6-R) consisted of 6 animals of either sex.

The required quantity of TCWE was weighed and triturated well in a mortar with a small quantity of vehicle (Type I Milli-Q water) until a homogenous suspension was formed. Thereafter, the entire quantity of the formulation was transferred into a measuring cylinder. A small quantity of vehicle was added to rinse the mortar and this was transferred into the measuring cylinder. The rinsing of mortar and pestle was repeated to ensure complete transfer of the contents to the measuring cylinder. Finally, the volume was made up to the required quantity with vehicle to get a desired concentration for different dose levels. The dose formulations were freshly prepared before dosing and continuously stirred using magnetic stirrer to maintain homogeneity during the dose administration.

The animals allocated to groups G1 and G5-R received vehicle whereas animals allocated to groups G2, G3 and G4/G6-R respectively received a daily dose of 100, 300 and 1000 mg/kg/body weight of TCWE for 28 consecutive days. TCWE/vehicle was administered by oral (gavage) route at a dose volume of 10 mL/kg with the concentrations of 10, 30 and 100 mg/mL for low, mid and high dose, respectively. The stock concentrations for each dose of TCWE were adjusted to keep the gavage volume constant at 10 mL/kg for all the doses. For example, a rat with weight of 250 g received 2.5 mL of 10, 30 and 100 mg/mL TCWE solution, for 100 mg/kg, 300 mg/kg and 1000 mg/kg doses, respectively. After day 28, dosing was withdrawn for recovery group animals, which were kept under observation for additional 14 days for delayed occurrence, or persistence of, or recovery from toxic effects. As per the Ayurvedic Pharmacopoeia of India, the recommended human dose range *T. cordifolia* is 3–6 g of raw herb in powder per day (API, Government of India, Ministry of Health and Family Welfare, Department of AYUSH, Part-I, Volume-I). However, the doses used in the current study were selected as per OECD 407 guidelines that recommend an upper limit of either 1000 mg/kg/day body weight or maximum feasible dose, whichever is higher.

#### 2.5.3 Morbidity, mortality observation and clinical sign monitoring

All animals were observed twice daily (morning and evening) for morbidity and mortality throughout the experimental period except on the day of terminal sacrifice, on which they were monitored in the morning only.

All animals were monitored before and after dose administration on day 1 and thereafter, once daily, throughout the experimental period. Detailed clinical examination was performed on day 1 and thereafter, on a weekly basis throughout the experimental period. The observed clinical signs included changes in skin, fur, eyes, mucous membranes, occurrence of secretions and excretions and autonomic activity, like, lacrimation, piloerection, pupil size, and unusual respiratory pattern. Changes in gait, posture and response to handling as well as the presence of clonic or tonic movements, stereotypes, like, excessive grooming and/or repetitive circling, or bizarre behaviour, such as, self-mutilation and/or walking backwards, were observed and recorded.

#### 2.5.4 Ophthalmological examination, functional observational battery (FOB)/neurobehavioral observation (NBO)

Ophthalmological examination was conducted for all animals prior to dosing, during acclimatization, and thereafter, on fourth week into dosing for control (G1), high dose group (G4) and recovery group (G5-R and G6-R) animals. Before ophthalmological examination, mydriasis was induced using 1% Tropicamide (AuroLab, Madurai, India; Batch No.: 9L906, Expiry Date: 30 November 2021, and Batch No.: 1B099, Expiry Date: 31 January 2023).

Functional Observational Battery (FOB)/neurobehavioral observation (NBO), included monitoring reactivity to stimuli, assessment of grip strength, hind limb foot splay and motor activity, was performed in the fourth week for main group animals and in the sixth week or the recovery group animals. Parameters, like, home cage measurements (body posture, activity/non-activity, gait, vocalization, clonic involuntary movement, tonic involuntary movement), hand held observations (reactivity to handling, lachrymation, salivation, piloerection, palpebral closure, crusty eyes, exopthalmus), open field activity mobility (ataxic gait, arousal, clonic convulsions, tonic convulsions, stereotyped behaviour, bizarre behaviour, number of defecations/minute, number of urine pools/minute), stimulus reactivity (approach response, touch response, click response, tail pinch, pupil response, eye blink response, forelimb response, hindlimb extension, righting reflex on the ground, air righting reflex, catalepsy, hindlimb extensor strength, hindlimb foot splay) neuromuscular observation (grip strength), pupil size and foot splay (landing hindlimb) were measured during FOB/NBO.

#### 2.5.5 Body weight and feed consumption measurement

Animals were weighed upon arrival at the test facility, on the day of randomization, at the start of treatment and weekly thereafter, till the end of experimental period. Fasting body weight of the animals were taken on the day of necropsy and used only to calculate the relative organ weight. Feed consumption of all animals was determined once weekly, during the treatment and recovery period.

#### 2.5.6 Clinical pathology investigations

##### 2.5.6.1 Blood collection

At the end of the treatment period, blood samples were collected from the live animals of the main groups (G1-G4) on day 29 and recovery groups (G5-R and G6-R) on day 43, prior to scheduled necropsies. Before scheduled sacrifice, animals were fasted overnight (12–14 h) with *ad libitum* access to drinking water. Blood was collected from retro-orbital sinus puncture with the help of a fine capillary tube under isoflurane anesthesia. Approximately 0.5 mL of blood was collected in each tube containing EDTA-K2/K3 (1.2 mg/mL of blood) and tri-sodium citrate (3.2% at ∼ 1:9 ratio of anticoagulant to blood) for analysis of hematological and coagulation parameters, respectively. Approximately 1.5–2 mL of blood was collected in tubes containing lithium heparin (12–30 IU/mL of blood) for analysis of clinical chemistry parameters. The blood samples were mixed gently by manual inversion 4–5 times, and kept in a coolant box, until centrifugation. Plasma was separated by centrifugation at 5,000–10,000 rpm for approximately 5 min, at 4°C ± 1°C. Plasma samples were stored at −70°C ± 10°C, immediately after separation till all the samples were further analyzed.

##### 2.5.6.2 Hematology and coagulation analysis

Hematological parameters, like, white blood cells, platelets, neutrophils, lymphocytes, monocytes, eosinophils, basophils, large unstained cells, red blood cell, reticulocyte, hemoglobin, mean corpuscular hemoglobin concentration, mean cellular hemoglobin concentration, hemoglobin distribution width, hematocrit, red cell distribution width, mean corpuscular volume, mean platelet volume, mean corpuscular hemoglobin and clonal hematopoiesis were determined using ADVIA-2120i Hematology System (Siemens, Malvern, PA, United States). Prothrombin time (PT) and activated partial thromboplastin time (APTT) analyses were carried out with plasma using Four Clot Analyzer (Robonik, Ambernath, Maharashtra, India).

##### 2.5.6.3 Clinical chemistry

In clinical chemistry analysis, total bilirubin, cholesterol, calcium, aspartate aminotransferase, alkaline phosphatase, triglyceride level, glucose, phosphate, alanine transaminase, creatinine, albumin, total protein, blood urea nitrogen, globulin and direct bilirubin were measured on Cobas c 111 Analyzer (Roche Diagnostics, Indianapolis, IN, United States). Sodium, potassium and chloride were analysed using NULYTE SMART Electrolyte Analyzer (Nirmal Medical Systems, New Delhi, India).

##### 2.5.6.4 Urinalysis

Prior to scheduled sacrifice, the study animals in treatment and recovery groups were housed in metabolic cages overnight, with *ad libitum* access to drinking (RO) water. Urine samples were collected for each animal and subjected to following qualitative and microscopic analyses.

Qualitative Tests. For qualitative analysis, volume, color and appearance of urine samples were noted through visual observations and recorded manually. Specific gravity, pH, total protein, glucose, blood, bilirubin and ketones were evaluated by using Eurocolor10 strips and analyzed on Urometer 720 Urine Chemistry Analyzer (Chicago, IL, United States).

Microscopic Examination. For microscopic examination, urine samples were centrifuged at approximately 2,500–3,000 rpm for 10 min and resulting sediments were taken and spread out on a glass slide. Sediments were evaluated microscopically for pus cells, epithelial cells, erythrocytes, crystals, bacteria, yeasts, casts and sperms.

#### 2.5.7 Pathology

##### 2.5.7.1 Necropsy and gross pathology

Prior to scheduled sacrifice, all animals were fasted overnight (12–14 h) and euthanized by Carbon dioxide (CO_2_) asphyxiation followed by exsanguination. During scheduled sacrifice, animals from different dose groups were segregated in such a way that approximately equal number of animals representing each dose group/sex were examined at similar time of the day. Complete necropsy examination was carried out on all animals and gross pathological findings (external and internal) were observed and recorded for each animal.

##### 2.5.7.2 Organ collection, weighing and fixation

On completion of the gross pathological examination, all the tissues and organs were collected from each animal, and processed for histology. Adrenals, brain (complete with cerebrum, cerebellum, medulla oblongata), epididymis, heart, kidneys, liver, ovaries with oviducts, prostrate, seminal vesicles with coagulating glands, spleen, testes, thymus and uterus with cervix were weighed. Adrenals, epididymis, kidneys, ovaries with oviducts and testes were weighed in pairs. Prostrate and seminal vesicles with coagulating glands were weighed after fixation. Eyes with optic nerves and Harderian glands were fixed in Davidson’s fluid, testes were fixed in modified Davidson’s fluid, and all other organs/tissues were fixed in 10% neutral buffered formalin. Relative organ weights were determined according to the following formula:

$$Relative\;organ\;weight\;\left(g\right)=\frac{Organ\;weight\;\left(g\right)}{Terminal\;body\;weight\;\left(g\right)}\times 100$$

##### 2.5.7.3 Histopathology

Required organs/tissues from all male and female animals of G1 (control) and G4 (high dose) groups and gross lesions from all groups and one animal of either sex from low (G2) and mid (G3) dose groups were trimmed, processed, embedded in paraffin blocks and sectioned (∼5 μ) in a microtome. Tissue slides were stained with Hematoxylin and Eosin, and subjected to microscopic evaluation. Bone marrow smears (using femur marrow) were prepared in duplicate at the time of necropsy. No treatment related effects were observed in hematology or during histopathology evaluation, hence bone marrow smears were not stained and evaluated. Histopathological evaluation of all the organs/tissues from all male and female animals from control (C1) and high dose (G4) groups and gross lesions were performed. Additionally, histopathological analysis was performed with organs/tissues from one animal of each sex in low (G2) and mid (G3) dose for representative photographs. No treatment related findings were observed in the high dose group (G4) animals, hence no histopathological evaluation was carried out for all the animals from lower dose and recovery groups.

### 2.6 Statistical analysis

Data was processed using Statistical Software SigmaPlot^®^ 12 (Systat, San Jose, California). The mean and standard deviation were calculated using the software and all data were summarized in tabular form. All continuous data (body weight, percent change in body weight with respect to day 1, feed consumption, haematology, clinical chemistry, absolute and relative organ weights, etc.) were checked for their normality. Statistical significance was analysed using one-way analysis of variance (ANOVA) followed by Dunnett’s post-hoc tests. In recovery groups, *t*-test was used for statistical analyses, followed by Mann Whitney’s test.

## 3 Results

### 3.1 Compositional analysis of TCWE

*T. cordifolia* is enriched in a huge variety of phytocompounds, primarily those belonging to terpenoid, alkaloid, lignan and steroid families (Sharma et al., 2019). Therefore, ultra high performance liquid chromatography (UHPLC) was used to detect the reported marker phytocompounds, magnoflorine and β-ecdysone in TCWE (Sharma et al., 2019) and establish its compositional fingerprint before the intended safety assessment. This would help in tracing the link between the phytoconstitutional composition and safety profile of the extract. Magnoflorine, besides being one of the marker phytocompounds of *T. cordifolia*, is associated with anti-nephro (Gupta and Sharma, 2011) and anti-hepatotoxic (Sharma and Dabur, 2015) effects. Likewise, β-ecdysone, along with remarkable anti-osteoporotic and bone-protective properties, is reported to be cytologically safe (Abiramasundari et al., 2018). Therefore, the phytoconstitutional integrity and traceability of TCWE safety was established through UHPLC with the marker compounds, magnoflorine and β-ecdysone. UHPLC analysis indeed confirmed the presence of magnoflorine (2.7 mg/g) and β-ecdysone (4.8 mg/g) in TCWE (Figure 1B).

### 3.2 Mortality and clinical signs

Different doses of TCWE (100, 300 and 1000 mg/kg/day) orally administered for consecutive 28 days to the study animals did not cause any mortality or morbidity among either sex. The animals from both sex in the dosage and recovery groups did not exhibit any treatment related clinical signs (Table 1). Detailed clinical monitoring confirmed the absence of all treatment-related clinical signs associated with Functional Observational Battery (FOB) and Neurobehavioral Observation (NBO).

**TABLE 1:** Clinical signs.

Sex	Groups (*n* = 6)	Treatment	TCWE dose (mg/kg BW)	Detailed clinical signs
Male	G1	Control	0	NAD
	G2	Low Dose	100	NAD
	G3	Mid Dose	300	NAD
	G4	High Dose	1000	NAD
	G5-R	Control Recovery	0	NAD
	G6-R	High Dose Recovery	1000	NAD
Female	G1	Control	0	NAD
	G2	Low Dose	100	NAD
	G3	Mid Dose	300	NAD
	G4	High Dose	1000	NAD
	G5-R	Control Recovery	0	NAD
	G6-R	High Dose Recovery	1000	NAD

NAD-No Abnormality Detected.

### 3.3 Effect of TCWE on the food consumption and body weight of the study animals

**FIGURE 2:**
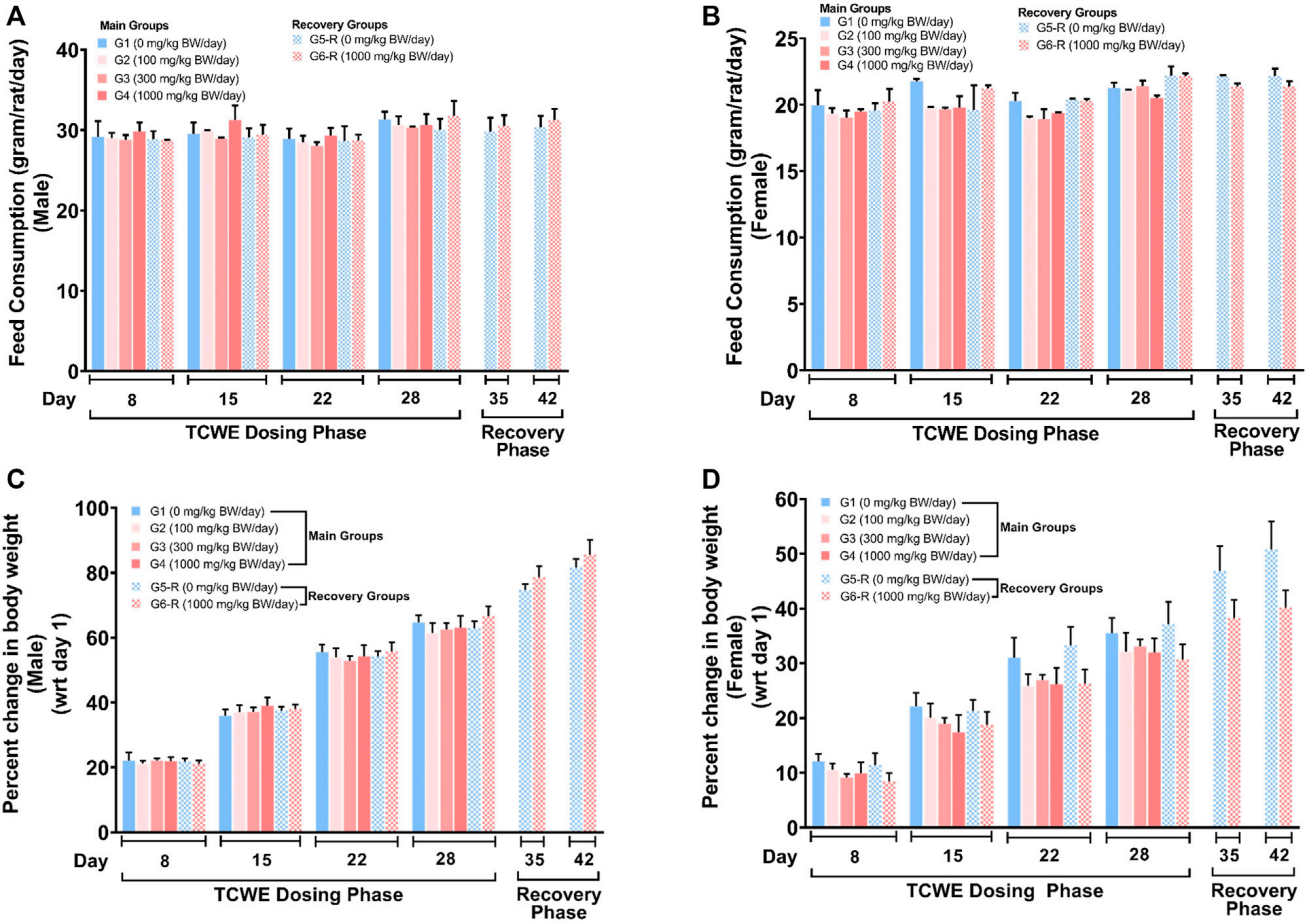
Effect of TCWE dosing and recovery on feed consumption and body weights of the study animals **(A, B)** Effect of TCWE dosing on the feed consumption (gram/rat/day) in male **(A)** and female **(B)** study animals belonging to different main groups (G1-G4) and recovery groups (G5-R and G6-R) shown as bar graphs over dosing (days 8, 15, 22 and 28) and recovery (days 35 and 42) phases. **(C, D)** Bar graphs showing the effect of TCWE dosing on body weights (as percent change *wrt* day 1) of male **(C)** and female **(D)** study animals from main (G1-G4) and recovery (G5-R and G6-R) groups over dosing (days 8, 15, 22 and 28) and recovery (days 35 and 42) phases.

The food consumption and body weights of the test animals of both sexes were measured once a week over the entire experimental regime (Figure 1A). For the animals in the main groups (G1- G4), these parameters were measured on days 8, 15, 22 and 28. For the animals in recovery groups (G5-R and G6-R), they were assessed further on days 35 and 42, in addition to those mentioned for the main groups. The weekly food consumption of the animals of both the sexes orally receiving 100 (G2), 300 (G3) and 1000 (G4, G6-R) mg/kg/day of TCWE were comparable to their counterparts belonging to the respective control groups (G1, G5-R) (Figures 2A, B). Consequently, the weekly percent changes in body weights *wrt* day 1 of the animals of either sex belonging to different groups did not show significant variation across the groups (Figures 2C, D).

### 3.4 Ophthalmological observations

The test animals were subjected to ophthalmological examination during quarantine before randomization and grouping on day 1 and at the end of dosing on day 28, for both the main and recovery groups (Figure 1A). No ophthalmological abnormalities were detected in the animals of either sex (Table 2).

**TABLE 2:** Opthalmoscopic signs.

Gender	Groups (*n* = 6)	Treatment	TCWE dose (mg/kg BW)	Ophthalmoscopic observation	
				Before treatment	After treatment
Male	G1	Control	0	NAD	NAD
	G2	Low Dose	100	NAD	NAD
	G3	Mid Dose	300	NAD	NAD
	G4	High Dose	1000	NAD	NAD
	G5-R	Control Recovery	0	NAD	NAD
	G6-R	High Dose Recovery	1000	NAD	NAD
Female	G1	Control	0	NAD	NAD
	G2	Low Dose	100	NAD	NAD
	G3	Mid Dose	300	NAD	NAD
	G4	High Dose	1000	NAD	NAD
	G5-R	Control Recovery	0	NAD	NAD
	G6-R	High Dose Recovery	1000	NAD	NAD

NAD-No Abnormality Detected.

### 3.5 Effect of TCWE on the hematological, coagulation and biochemical parameters of the study animals

After terminal sacrifice of the study animals of both the sexes on day 29 from main groups (G1—G4) and on day 43 from recovery groups (G5-R, G6-R), blood was collected and subjected to hematological, coagulation and biochemical analyses (Figure 1A).

#### 3.5.1 Hematological and coagulation analyses

Neutrophil counts (10^3^/μL) in the male study animals from the recovery group G6-R, that received 1000 mg/kg/day of TCWE orally for 28 days, were high [2.73(±0.77)*10^3^/μL; *p* < 0.05] compared to the corresponding control group, G5-R [1.83(±0.51)*10^3^/μL]. Although statistically significant, this increase was an incidental finding with no associated dose response as evident from the readings from different main groups [G1: 1.9(±0.46) *10^3^/μL; G2: 2.28(±0.68) *10^3^/μL; G3: 2.26(±0.66) *10^3^/μL and G4: 1.96(±0.45) *10^3^/μL]. Furthermore, lack of corroboration between the readings from groups, G4 [1.96(±0.45) *10^3^/μL] and G6-R [2.73(±0.77)*10^3^/μL] confirms that this increase in the neutrophil count in the high dose recovery group is not related to TCWE administration. As expected this readout was reflected in the percent neutrophil counts [Neut (%)], since, this value was generated from the absolute neutrophil counts (Table 3).

**TABLE 3:** Hematology and coagulation analysis.

Group (*n* = 6)	G1	G2	G3	G4	G5-R	G6-R	G1	G2	G3	G4	G5-R	G6-R
**Treatment**	**Control**	**Low dose**	**Mid dose**	**High dose**	**Control Recovery**	**High Dose Recovery**	**Control**	**Low dose**	**Mid dose**	**High dose**	**Control Recovery**	**High Dose Recovery**
**TCWE Dose (mg/kg BW)**	**0**	**100**	**300**	**1000**	**0**	**1000**	**0**	**100**	**300**	**1000**	**0**	**1000**
**Male**							**Female**					
**WBC (10** ^**3**^ **/µL)**	10.93 ± 2.53	10.44 ± 1.6	10.84 ± 2.42	10.47 ± 3.36	10.38 ± 1.53	12.39 ± 3.37	8.2 ± 1.54	8.46 ± 2.09	9.74 ± 2.66	10.7 ± 2.57	9.48 ± 2.1	8.19 ± 1.37
**Plat (10** ^**3**^ **/µL)**	908.5 ± 205.66	876 ± 247.2	792.17 ± 274.91	1131.17 ± 149.41	953.5 ± 188.92	1003.67 ± 187.75	921.83 ± 155.28	951.5 ± 140.35	876.67 ± 222.54	925.33 ± 153.6	998.67 ± 122.84	1003.67 ± 176.42
**Neut (10** ^**3**^ **/µL)**	1.9 ± 0.46	2.28 ± 0.68	2.26 ± 0.66	1.96 ± 0.45	1.83 ± 0.51	2.73↑±0.77	1.43 ± 0.44	2.12 ± 0.78	1.74 ± 0.5	1.44 ± 0.3	1.45 ± 0.22	1.56 ± 0.41
**Lym (10** ^**3**^ **/µL)**	8.52 ± 2.2	7.61 ± 1.92	8.01 ± 1.76	7.98 ± 3.13	8.12 ± 1.16	9.11 ± 2.7	6.24 ± 1.9	5.89 ± 1.68	7.5 ± 2.5	8.86 ± 2.49	7.69 ± 2.05	6.34 ± 1.32
**Mon (10** ^**3**^ **/µL)**	0.27 ± 0.12	0.31 ± 0.13	0.31 ± 0.15	0.31 ± 0.06	0.2 ± 0.07	0.24 ± 0.11	0.19 ± 0.08	0.23 ± 0.04	0.23 ± 0.1	0.17 ± 0.06	0.13 ± 0.03	0.1 ± 0.03
**Eos (10** ^**3**^ **/µL)**	0.14 ± 0.08	0.11 ± 0.03	0.15 ± 0.06	0.11 ± 0.05	0.12 ± 0.05	0.14 ± 0.04	0.13 ± 0.03	0.12 ± 0.05	0.16 ± 0.05	0.11 ± 0.02	0.11 ± 0.03	0.1 ± 0.06
**Bas (10** ^**3**^ **/µL)**	0.03 ± 0.01	0.06 ± 0.03	0.04 ± 0.02	0.04 ± 0.01	0.03 ± 0.01	0.04 ± 0.02	0.02 ± 0.01	0.02 ± 0.01	0.02 ± 0.01	0.02 ± 0.01	0.03 ± 0.02	0.02 ± 0.01
**LUC (10** ^**3**^ **/µL)**	0.07 ± 0.04	0.07 ± 0.02	0.08 ± 0.03	0.07 ± 0.02	0.09 ± 0.03	0.13 ± 0.05	0.2 ± 0.27	0.09 ± 0.03	0.08 ± 0.04	0.1 ± 0.04	0.07 ± 0.04	0.07 ± 0.03
**RBC (10** ^**6**^ **/µL)**	7.62 ± 0.3	8.03 ± 0.38	7.87 ± 0.52	7.79 ± 0.54	8.07 ± 0.23	7.94 ± 0.39	7.6 ± 0.39	7.57 ± 0.29	7.58 ± 0.34	7.57 ± 0.23	7.63 ± 0.3	7.86 ± 0.39
**Retic (10** ^**9**^ **/L)**	178.13 ± 25.35	165.3 ± 64.84	156.13 ± 24.21	167.73 ± 52.38	213.95 ± 32.51	185.8 ± 27.22	196.63 ± 29.04	184.77 ± 38.31	144.93 ± 22.71	173.92 ± 55.11	210.8 ± 31.9	207.38 ± 44.95
**Hb (g/dL)**	19.18 ± 0.68	20.08 ± 1.14	19.15 ± 1.74	19.35 ± 1.79	18.45 ± 0.38	18.55 ± 0.73	19.32 ± 1.02	19.23 ± 1.04	19.5 ± 0.86	19.05 ± 0.49	17.92 ± 0.65	18.15 ± 0.89
**MCHC (g/dL)**	38.97 ± 0.87	39.33 ± 1.02	37.87 ± 1.42	38.17 ± 1.05	39.77 ± 0.5	39.92 ± 0.38	41.15 ± 1.14	42.13 ± 1.44	42.73 ± 0.83	41.85 ± 0.65	40.4 ± 0.77	40.3 ± 0.85
**CHCM (g/dL)**	29.47 ± 0.38	29.78 ± 0.16	29.53 ± 0.42	29.7 ± 0.59	31.38 ± 0.31	31.37 ± 0.25	31.25 ± 0.1	31.2 ± 0.46	31.15 ± 0.48	31.22 ± 0.43	31.9 ± 0.32	31.63 ± 0.44
**HDW (g/dL)**	2.22 ± 0.12	2.18 ± 0.09	2.14 ± 0.12	2.17 ± 0.11	2.23 ± 0.12	2.18 ± 0.19	2.07 ± 0.08	2.08 ± 0.09	1.96↓±0.02	1.94↓±0.09	2.07 ± 0.1	1.96 ± 0.15
**HCT (%)**	49.2 ± 1.38	51.05 ± 3.16	50.48 ± 3.33	50.65 ± 3.97	46.38 ± 0.97	46.48 ± 1.75	46.95 ± 2.22	45.62 ± 1.4	45.65 ± 1.71	45.52 ± 1.1	44.3 ± 1.47	45.08 ± 1.58
**Neut (%)**	17.7 ± 3.68	22.32 ± 7.53	20.82 ± 2.67	19.67 ± 5.78	17.38 ± 3.32	22.10↑±2.91	18.38 ± 7.89	25.15 ± 7.7	18.87 ± 6.54	14.17 ± 4.19	15.88 ± 4	19.43 ± 5.87
**Lym (%)**	77.63 ± 3.89	72.28 ± 9	74 ± 2.55	74.97 ± 6.97	78.4 ± 4.18	73.33↓±3.45	74.9 ± 11.86	69.15 ± 7.79	75.92 ± 6.85	82.15 ± 4.32	80.28 ± 4.63	77.05 ± 5.78
**Mon (%)**	2.5 ± 1.06	3.08 ± 1.29	2.78 ± 1.38	3.17 ± 1.09	1.95 ± 0.71	2.02 ± 0.97	2.4 ± 1.22	2.88 ± 1.08	2.37 ± 0.76	1.38 ± 0.39	1.45 ± 0.47	1.17 ± 0.37
**Eos (%)**	1.27 ± 0.52	1.08 ± 0.31	1.37 ± 0.55	1.1 ± 0.45	1.13 ± 0.51	1.25 ± 0.48	1.58 ± 0.41	1.52 ± 0.85	1.73 ± 0.52	1 ± 0.5	1.3 ± 0.53	1.27 ± 0.77
**Bas (%)**	0.25 ± 0.1	0.52 ± 0.34	0.35 ± 0.19	0.43 ± 0.19	0.27 ± 0.05	0.33 ± 0.12	0.2 ± 0.13	0.2 ± 0.06	0.22 ± 0.12	0.27 ± 0.28	0.33 ± 0.16	0.26 ± 0.11
**Retic (%)**	2.37 ± 0.29	2.61 ± 0.12	2.02 ± 0.32	2.21 ± 0.65	2.65 ± 0.38	2.35 ± 0.41	2.58 ± 0.3	2.45 ± 0.55	1.92 ± 0.33	2.28 ± 0.67	2.76 ± 0.4	2.62 ± 0.46
**RDW (%)**	10.78 ± 0.35	10.65 ± 0.34	10.57 ± 0.2	10.48 ± 0.24	10.77±	10.7 ± 0.28	10.17 ± 0.19	9.92 ± 0.31	9.77↓±0.2	9.83 ± 0.24	10.38 ± 0.21	10.07 ± 0.28
**LUC (%)**	0.65 ± 0.34	0.7 ± 0.2	0.7 ± 0.32	0.67 ± 0.16	0.85 ± 0.36	0.98 ± 0.21	2.5 ± 3.59	1.12 ± 0.34	0.88 ± 0.35	0.9 ± 0.21	0.77 ± 0.34	0.8 ± 0.38
**MCV (fL)**	64.6 ± 1.29	63.55 ± 1.56	64.12 ± 1.19	65.07 ± 1.92	57.53 ± 1.33	58.6 ± 1.2	61.85 ± 2.11	60.33 ± 1.24	60.28 ± 0.83	60.15 ± 0.79	58.07 ± 1.17	57.37 ± 1.29
**MPV (fL)**	7.7 ± 0.4	7.87 ± 0.34	8.17 ± 0.42	7.68 ± 0.19	6.47 ± 0.22	6.7 ± 0.33	6.83 ± 0.16	6.97 ± 0.27	7.03 ± 0.36	7.02 ± 0.37	6.98 ± 0.31	6.88 ± 0.17
**MCH (pg)**	25.18 ± 0.84	25 ± 0.53	24.28 ± 0.74	24.85 ± 1.3	22.9 ± 0.8	23.38 ± 0.5	25.45 ± 1.37	25.43 ± 1.17	25.78 ± 0.78	25.17 ± 0.62	23.45 ± 0.54	23.1 ± 0.79
**CH (pg)**	19 ± 0.42	18.9 ± 0.41	18.92 ± 0.1	19.28 ± 0.63	18.03 ± 0.58	18.35 ± 0.44	19.28 ± 0.64	18.8 ± 0.49	18.77 ± 0.56	18.72 ± 0.29	18.48 ± 0.36	18.1 ± 0.41
**PT (sec)**	24.62 ± 2.17	24.18 ± 1.2	23.83 ± 2.62	22.85 ± 1.18	23.16 ± 4.1	23.19 ± 2.95	28.8 ± 2.91	25.06↓±0.89	25.03↓±2.1	23.57↓±1.03	24.56 ± 1.89	23.95 ± 3.03
**APTT (sec)**	35.83 ± 2.28	25.25↓ ± 3.26	28.04↓ ± 5.04	22.18↓±4.16	21.23 ± 3.33	21.48 ± 2.85	26.33 ± 1.99	22.37 ± 3.79	21.83↓±2.97	25.63 ± 2.3	20.69 ± 2.5	18.89 ± 3.21

Values are Mean ± SD, n = Number of animals in each group, ↑significantly (*p* < 0.05) higher than control (G1) group, ↓significantly (*p* < 0.05) lower than control (G1) group.

TCWE, *Tinospora cordifolia* water extract; WBC, white blood cell; Plat, Platelet; Neut, Neutrophil; Lym, Lymphocyte; Mon, Monocyte; Eos, Eosinophil; Bas, Basophil; LUC, large unstained cell; RBC, red blood cell; Retic, Reticulocyte; Hb, Hemoglobin; MCHC, mean corpuscular hemoglobin concentration; CHCM, cellular hemoglobin concentration mean; HWD, hemoglobin distribution width; HCT, hemotocrit; RDW, red cell distribution width; MCV, mean corpuscular volume; MPV, mean platelet volume; MCH, mean corpuscular hemoglobin; CH, clonal hematopoiesis; PT, prothrombin Time; APTT, activated partial thromboplastin time.

Bold values represent different TCWE doses, as mentioned in either the column or the row heading.

The coagulation parameter APTT showed that blood clotting was slower in the male study animals from groups G2, G3 and G4 receiving 100, 300 and 1000 mg/kg/day of TCWE orally, when compared to their counterparts in the vehicle control group G1. This was evident from the increased (*p* < 0.05) blood clotting time reported as APTT (sec) readouts [25.25 (±3.26), 28.04(±5.04) and 22.18(±4.16) sec, respectively in G2, G3 and G4 compared to 35.83(±2.28) sec in G1]. Clearly, there was no noticeable dose-dependency in this observation, making it another incidental finding, with no co-relation with the TCWE administration (Table 3). A similar observation regarding APTT was made for the female study animals belonging to G3 [21.83(±2.97) sec *versus* 26.33(±1.99) sec in G1] (Table 3). Since, there was no observed dose-dependency, this finding, as above is also incidental. The female study animals belonging to the main groups, G2—G4 also exhibited statistically significant (*p* < 0.05) decrease [25.06(±0.89), 25.03(±2.1) and 23.57(±1.03) sec, respectively in G2, G3 and G4 versus 28.8(±2.91) sec in corresponding G1] in PT, another coagulation parameter that measures the time taken for a clot to form in a blood sample (Table 3). The change was not a dose response as evident from variation between the PT readouts of two technically identical control groups, G1 (28.8 ± 2.91 s) and G5-R (24.56 ± 1.89).

#### 3.5.2 Biochemical analysis

Serum albumin [Alb (g/dL)] was found to be increased [4.68(±0.01) g/dL *versus* 4.45(±0.22) g/dL in corresponding control group G1] in male study animals from the main group G3, which received 300 mg/kg/day of TCWE orally for 28 days. However, this increase had no dose-dependency, making it an incidental finding with no co-relatable effect from the TCWE dosage (Table 4). The blood urea nitrogen [BUN (mg/dL)] content of the serum in the male study animals of the recovery group G6-R was significantly reduced [12.63(±0.96) mg/dL] as compared to the corresponding control group G [14.06(±2.48) mg/dL]. In the female study animals of main group G2 (receiving 100 mg/kg/day of TCWE orally for 28 days), BUN was increased [18.11(±2.13) mg/dL] in comparison to the corresponding control group G1 [14.45(±1.00) mg/dL]. However, due to lack of dose-dependency, these observations are considered to be incidental findings unrelated to TCWE dosage (Table 4). Two other incidental deviations, with no dose dependent co-relation whatsoever, in the female study animals were observed in case of increased urea [38.76(±4.57) in G2 *versus* 30.91(±2.15) mg/dL in G1] and decreased glucose (Glu) [114.45(±10.05) in G6-R *versus* 127.85(±9.35) mg/dL in G5-R] (Table 4).

**TABLE 4:** Clinical chemistry.

Group (*n* = 6)	Treatment	TCWE dose (mg/kg BW)	T. Bil (mg/dL)	CHOL (mg/dL)	Ca (mg/dL)	Urea (mg/dL)	AST (U/L)	ALP (U/L)	TGL (mg/dL)	Glu (mg/dL)	Phos (mg/dL)	ALT (U/L)	Creat (mg/dL)	Alb (g/dL)	T.Pro (g/dL)	BUN (mg/dL)	Glob (g/dL)	Na (mmol/L)	K (mmol/L)	Cl (mmol/L)	D.Bil (mg/dL)
Male																					
**G1**	**Control**	**0**	0.06	48.21	10.54	30.08	78.23	142.5	39.32	134.51	7.81	29.79	0.36	4.45	6.09	14.06	1.64	138.43	4.01	100.7	0.05
			±	±	±	±	±	±	±	±	±	±	±	±	±	±	±	±	±	±	±
			0.03	6.99	0.32	5.3	12.5	24.09	7.78	18.69	0.63	5.04	0.06	0.22	0.25	2.48	0.3	0.71	0.37	0.84	0.01
**G2**	**Low dose**	**100**	0.04	56.81	10.38	31.74	66.92	125.2	41.43	141.74	7.08	24.13	0.37	4.49	6.17	14.83	1.68	138.1	3.83	100.53	0.05
			±	±	±	±	±	±	±	±	±	±	±	±	±	±	±	±	±	±	±
			0.06	10.78	0.28	4.08	6.45	25.38	12.61	15.69	0.31	5.07	0.04	0.14	0.15	1.9	0.1	1.04	0.22	1.16	0.02
**G3**	**Mid dose**	**300**	0.06	45.94	10.77	30.19	77.47	155	36.23	138.89	7.5	29.86	0.33	4.68↑	6.19	14.11	1.52	138.32	4.02	101.2	0.05
			±	±	±	±	±	±	±	±	±	±	±	±	±	±	±	±	±	±	±
			0.03	10.74	0.43	3.36	8.29	27.32	5.19	23.07	0.6	7.73	0.02	0.1	0.17	1.57	0.13	0.67	0.31	1.3	0.03
**G4**	**High dose**	**1000**	0.07	43.63	11.11	31.17	70.33	129.4	43.73	129.52	7.19	24.22	0.3	4.61	7.01	14.57	2.4	138	4	100.83	0.06
			±	±	±	±	±	±	±	±	±	±	±	±	±	±	±	±	±	±	±
			0.06	9.38	0.95	6.42	10.05	21.77	21.23	22.75	0.78	2.27	0.04	0.12	2.38	3	2.36	0.78	0.17	1.26	0.02
**G5-R**	**Control Recovery**	**0**	0.09	46.21	10.35	30.04	71.95	107.4	44.98	121.85	7.16	26.75	0.35	4.65	6.6	14.04	1.95	140.25	3.71	102.77	0.04
			±	±	±	±	±	±	±	±	±	±	±	±	±	±	±	±	±	±	±
			0.05	12.93	0.31	2.13	11.87	26.19	18.23	22.81	0.42	4.47	0.07	0.21	0.27	0.99	0.23	0.56	0.23	0.74	0.03
**G6-R**	**High Dose Recovery**	**1000**	0.07	42.95	10.39	27.02↓	64.88	111.6	32.61	113.55	6.86	23.8	0.35	4.77	6.83	12.63↓	2.05	140.05	3.64	102.92	0.05
			±	±	±	±	±	±	±	±	±	±	±	±	±	±	±	±	±	±	±
			0.06	7.16	0.23	2.06	3.78	19.77	9.65	8.61	0.36	2.86	0.04	0.19	0.18	0.96	0.09	0.82	0.18	0.86	0.02
**Female**																					
**G1**	**Control**	**0**	0.01	58.71	10.58	30.91	76.08	86.71	40.05	100.43	7.21	25.75	0.41	5.1	6.44	14.45	1.33	139.77	3.6	105.88	0.06
			±	±	±	±	±	±	±	±	±	±	±	±	±	±	±	±	±	±	±
			0.02	6.32	0.24	2.15	4.86	9.41	6.23	8.66	0.23	3.63	0.06	0.22	0.14	1.00	0.18	0.29	0.22	0.68	0.02
**G2**	**Low dose**	**100**	0.03	57.39	10.5	38.76↑	78.5	78.69	35.2	103.59	6.5	26.51	0.43	5.23	6.56	18.11↑	1.33	139.67	3.46	106	0.05
			±	±	±	±	±	±	±	±	±	±	±	±	±	±	±	±	±	±	±
			0.04	9.8	0.2	4.57	10.83	18.04	6.8	22.44	0.5	9.1	0.09	0.23	0.27	2.13	0.2	0.45	0.41	1.02	0.01
**G3**	**Mid dose**	**300**	0.02	59.91	10.79	36.83	74.97	84.07	40.38	103.23	6.84	27.06	0.4	5.36	6.69	17.21	1.34	139.33	3.61	105.87	0.05
			±	±	±	±	±	±	±	±	±	±	±	±	±	±	±	±	±	±	±
			0.03	11.46	0.29	5.09	16.62	17.04	16.36	25.17	1.08	4.86	0.07	0.29	0.15	2.38	0.24	0.79	0.47	0.81	0.01
**G4**	**High dose**	**1000**	0.01	51.9	10.83	36.84	76.32	86.74	30.34	99.36	7.39	21.87	0.39	5.19	6.55	17.22	1.36	139.3	3.6	105.58	0.05
			±	±	±	±	±	±	±	±	±	±	±	±	±	±	±	±	±	±	±
			0.02	13.84	0.47	4.04	9.51	21.57	3.25	18.04	1.16	3.22	0.05	0.42	0.21	1.89	0.32	0.68	0.41	1.06	0.02
**G5-R**	**Control Recovery**	**0**	0.05	53.12	10.43	32.46	74.78	66.65	31.51	127.85	5.62	23.4	0.4	5.23	6.91	15.17	1.67	139.08	3.38	104.62	0.04
			±	±	±	±	±	±	±	±	±	±	±	±	±	±	±	±	±	±	±
			0.05	7.08	0.18	5.66	12.1	10.8	5.12	9.35	0.42	3.49	0.07	0.25	0.18	2.64	0.23	0.81	0.14	0.62	0.03
**G6-R**	**High Dose Recovery**	**1000**	0.06	52.21	10.39	38.3	81.05	66.05	31.09	114.45↓	5.74	21.53	0.41	5.38	6.95	17.89	1.57	139.15	3.48	105.05	0.03
			±	±	±	±	±	±	±	±	±	±	±	±	±	±	±	±	±	±	±
			0.05	6.52	0.15	7.94	23.11	18.94	9.23	10.05	0.77	3.77	0.05	0.25	0.21	3.71	0.38	1.13	0.24	1.41	0.01

Values are Mean ± SD, n = Number of animals in each group, ↑significantly (*p* < 0.05) higher than control (G1) group, ↓significantly (*p* < 0.05) lower than control (G1) group.

TCWE, *Tinospora cordifolia* water extract; T. Bil, Total Bilirubin; CHOL, cholesterol; Ca, Calcium; AST, aspartate aminotransferase; ALP, alkaline phosphatase; TGL, Triglyceride level; Glu, Glucose; Phos, Phosphate; ALT, alanine transaminase; Creat, Creatinine; Alb, Albumin; T. Pro, Total protein; BUN, blood urea nitrogen; Glob, Globulin; Na, Sodium; K, potassium; Cl, Chlorine; D. Bil, Direct bilirubin.

Bold values represent different TCWE doses, as mentioned in either the column or the row heading.

#### 3.5.3 Urine analysis to determine adverse effects of TCWE repeated dosing

Urine analysis was conducted as end-point assessments in the male and female study animals of main groups (G1—G4) on day 29 and recovery groups (G5-R and G6-R) on day 43 (Figure 1A). The 28 days repeated oral dosing of TCWE at different concentrations (100, 300 and 1000 mg/kg/day) followed by 14-day recovery did not exhibit any adverse effects on the parameters of urine analysis (Table 5; Table 6).

**TABLE 5:** Urine analysis (physical attributes).

Group (*n* = 6)	Treatment	TCWEDose (mg/kg BW)	Volume (mL)	Colour	Appearance	Glucose	Bilirubin	Pus cells	Epithelial cells	RBCs	Cast	Crystal	Bacteria	Yeast	Sperms
Male															
**G1**	**Control**	**0**	12.92 ± 0.66	Pale yellowish	Clear	Absent	Absent	Absent	Absent	Absent	Absent	Present	Absent	Absent	Present
**G2**	**Low dose**	**100**	12.58 ± 0.8	Pale yellowish	Clear	Absent	Absent	Absent	Absent	Absent	Absent	Present	Absent	Absent	Present
**G3**	**Mid dose**	**300**	12.67 ± 0.61	Pale yellowish	Clear	Absent	Absent	Absent	Absent	Absent	Absent	Present	Absent	Absent	Present
**G4**	**High dose**	**1000**	12.42 ± 0.38	Pale yellowish	Clear	Absent	Absent	Absent	Absent	Absent	Absent	Present	Absent	Absent	Present
**G5-R**	**Control Recovery**	**0**	10.5 ± 0.45	Pale yellowish	Clear	Absent	Absent	Absent	Absent	Absent	Absent	Present	Absent	Absent	Present
**G6-R**	**High Dose Recovery**	**1000**	10.17 ± 0.52	Pale yellowish	Clear	Absent	Absent	Absent	Absent	Absent	Absent	Present	Absent	Absent	Present
**Female**															
**G1**	**Control**	**0**	11.42 ± 0.38	Pale yellowish	Clear	Absent	Absent	Absent	Absent	Absent	Absent	Present	Absent	Absent	Absent
**G2**	**Low dose**	**100**	11.33 ± 0.41	Pale yellowish	Clear	Absent	Absent	Absent	Absent	Absent	Absent	Present	Absent	Absent	Absent
**G3**	**Mid dose**	**300**	11.32 ± 0.43	Pale yellowish	Clear	Absent	Absent	Absent	Absent	Absent	Absent	Present	Absent	Absent	Absent
**G4**	**High dose**	**1000**	11.03 ± 0.24	Pale yellowish	Clear	Absent	Absent	Absent	Absent	Absent	Absent	Present	Absent	Absent	Absent
**G5-R**	**Control Recovery**	**0**	10 ± 0.45	Pale yellowish	Clear	Absent	Absent	Absent	Absent	Absent	Absent	Present	Absent	Absent	Absent
**G6-R**	**High Dose Recovery**	**1000**	9.83 ± 0.41	Pale yellowish	Clear	Absent	Absent	Absent	Absent	Absent	Absent	Present	Absent	Absent	Absent

Values are Mean ± SD, n = Number of animals.

Bold values represent different TCWE doses, as mentioned in either the column or the row heading.

**TABLE 6:** Urine analysis (chemical composition).

Group (*n* = 6)	Treatment	TCWE dose (mg/kg BW)	Bilirubin (mg/dL)	Ketone (mg/dL)	Specific gravity	Blood (RBCs/μL)	pH	Protein (mg/dL)	Urobilinogen (mg/dL)	Glucose (mg/dL)	Leucocyte (WBCs/μL)	Nitrite (mg/dL)
Male												
**G1**	**Control**	**0**	0	14.17 ± 17.72	1.02 ± 0	0	7.75 ± 0.42	16.67 ± 15.06	0.1 ± 0	0	6.67 ± 5.16	0
**G2**	**Low dose**	**100**	0	13.33 ± 18.35	1.02 ± 0	0	7.92 ± 0.2	6.67 ± 5.16	0.1 ± 0	0	8.33 ± 4.08	0
**G3**	**Mid dose**	**300**	0	22.5 ± 21.39	1.02 ± 0	0	7.25 ± 0.27	10 ± 10.95	0.1 ± 0	0	6.67 ± 5.16	0
**G4**	**High dose**	**1000**	0	20 ± 23.45	1.02 ± 0	0	7.33 ± 0.61	3.33 ± 5.16	0.1 ± 0	0	2 ± 4.47	0
**G5-R**	**Control Recovery**	**0**	0	14.17 ± 18	1.02 ± 0	0.83 ± 2.04	8 ± 0	36.67 ± 33.27	0.1 ± 0	0	0	0
**G6-R**	**High Dose Recovery**	**1000**	0	20 ± 23.66	1.01 ± 0	8.33 ± 20.41	8 ± 0.55	18.33 ± 40.21	0.1 ± 0	0	0	0
**Female**												
**G1**	**Control**	**0**	0	2.5 ± 4.18	1.02 ± 0	0	7 ± 0.55	3.33 ± 5.16	0.1 ± 0	0	0	0
**G2**	**Low dose**	**100**	0	0	1.01 ± 0	0	7.75 ± 0.61	0	0.1 ± 0	0	0	0
**G3**	**Mid dose**	**300**	0	0	1.02 ± 0	0	7.33 ± 0.41	0	0.1 ± 0	0	0	0
**G4**	**High dose**	**1000**	0	0	1.02 ± 0	0	7.33 ± 0.52	0	0.1 ± 0	0	0	0
**G5-R**	**Control Recovery**	**0**	0	0	1.02 ± 0	0	7.83 ± 0.52	3.33 ± 5.16	0	0	0	0
**G6-R**	**High Dose Recovery**	**1000**	0	0	1.02 ± 0.01	0	7.67 ± 0.68	0	0	0	0	0

Values are Mean ± SD, n = Number of animals, ↑significantly (*p* < 0.05) Higher than control (G1) group, ↓significantly (*p* < 0.05) lower than control (G1) group.

Bold values represent different TCWE doses, as mentioned in either the column or the row heading.

#### 3.5.4 Necropsy and histopathology to evaluate the effects of TCWE on different organs

##### 3.5.4.1 Necropsy

Necropsy was conducted on the study animals of both the sexes from main (G1—G4) as well as recovery (G5-R and G6-R) groups after terminal sacrifices on days 29 and 43, respectively (Figure 1A). Weights of individual organs (relative to the whole body weight) of animals in the main and recovery groups receiving TCWE dosing were comparable to their respective control groups, G1 and G5-R (Table 7; Table 8).

**TABLE 7:** Relative Organ Weight (*wrt* body weight) (Male).

Group (*n* = 6)	Treatment	TCWE dose (mg/kg BW)	Terminal body weight	Adrenal	Thymus	Spleen	Epididymides	Testes	Brain	Heart	Kidneys	Liver	Prostate	Seminal vessicle
**G1**	**Control**	**0**	363.69	0.0163	0.1672	0.1936	0.2881	0.8891	0.557	0.4029	0.792	3.6263	0.2907	0.3301
			±	±	±	±	±	±	±	±	±	±	±	±
			38.41	0.0017	0.0326	0.0147	0.0283	0.0931	0.0582	0.0493	0.0739	0.3181	0.0217	0.0433
**G2**	**Low dose**	**100**	372.06	0.0153	0.138	0.2164	0.2899	0.8604	0.5636	0.3753	0.7658	3.5464	0.285	0.3087
			±	±	±	±	±	±	±	±	±	±	±	±
			27.79	0.001	0.0197	0.0512	0.0233	0.0786	0.0482	0.0247	0.0442	0.2361	0.0446	0.0291
**G3**	**Mid dose**	**300**	366.59	0.0144	0.1444	0.1929	0.2829	0.8476	0.5584	0.3768	0.801	3.6193	0.2472	0.3078
			±	±	±	±	±	±	±	±	±	±	±	±
			12.87	0.0016	0.0077	0.027	0.0264	0.0593	0.0283	0.0178	0.0603	0.2704	0.0291	0.0299
**G4**	**High dose**	**1000**	371.85	0.0157	0.1405	0.2015	0.3021	0.837	0.5677	0.3961	0.7838	3.8929	0.2597	0.3376
			±	±	±	±	±	±	±	±	±	±	±	±
			31.13	0.0013	0.019	0.0281	0.0154	0.0509	0.037	0.0383	0.0193	0.0965	0.0268	0.0237

Values are Mean ± SD, n = Number of animals, ↑significantly (*p* < 0.05) Higher than control (G1) group, ↓significantly (*p* < 0.05) lower than control (G1) group.

Bold values represent different TCWE doses, as mentioned in either the column or the row heading.

**TABLE 8:** Relative Organ Weight (*wrt* body weight) (Female).

Group (*n* = 6)	Treatment	TCWE dose (mg/kg BW)	Terminal body weight	Adrenals	Thymus	Spleen	Uterus/Cervix	Ovaries	Brain	Heart	Kidneys	Liver
**G1**	**Control**	**0**	227.44	0.0275	0.207	0.2208	0.286	0.0669	0.8525	0.348	0.8204	3.7656
			±	±	±	±	±	±	±	±	±	±
			19.16	0.0032	0.0161	0.0377	0.1033	0.0111	0.0779	0.1486	0.0649	0.2418
**G2**	**Low dose**	**100**	222.54	0.03	0.1892	0.2253	0.2904	0.0622	0.8339	0.3956	0.8253	3.6635
			±	±	±	±	±	±	±	±	±	±
			12.43	0.0048	0.0423	0.0405	0.0839	0.0041	0.0242	0.035	0.0624	0.1565
**G3**	**Mid dose**	**300**	222.21	0.028	0.2027	0.1998	0.1936	0.0651	0.8844	0.4099	0.7861	3.5935
			±	±	±	±	±	±	±	±	±	±
			9.6	0.0026	0.027	0.0261	0.0136	0.0099	0.0375	0.0291	0.0655	0.2121
**G4**	**High dose**	**1000**	216.61	0.0284	0.2044	0.1822	0.2387	0.0615	0.9039	0.4281	0.8252	3.7232
			±	±	±	±	±	±	±	±	±	±
			10.25	0.0016	0.0457	0.0147	0.1016	0.0042	0.0542	0.0276	0.071	0.2217

Values are Mean ± SD, n = Number of animals, ↑significantly (*p* < 0.05) Higher than control (G1) group, ↓significantly (*p* < 0.05) lower than control (G1) group.

Bold values represent different TCWE doses, as mentioned in either the column or the row heading.

##### 3.5.4.2 Gross pathology

Gross pathological observations were conducted on all the study animals belonging to either sex across main (G1—G4) and recovery (G5-R and G6-R) groups, after terminal sacrifices on days 29 and 43, respectively (Figure 1A). External and internal examination of the terminally sacrificed male study animals from vehicle control (G1) and TCWE dosing groups (G2, G3 and G4) did not reveal any macroscopic or gross pathological findings. Similarly, terminally sacrificed female study animals belonging to vehicle control (G1) and TCWE dosing groups (G3 and G4) did not reveal any macroscopic or gross pathological findings. Therefore, from gross pathological observations, it could be concluded that no treatment related gross (external and internal) pathological findings were noted at 1000 mg/kg dose in either sex. However, one female [animal no: 31 from G2 (100 mg/kg/day)] and two male study animals (animal no: 54 from control G5-R and animal no: 58 from G6-R (1000 mg/kg/day) showed mild reduction in the size of thymus. Sections of thymus from these study animals were evaluated microscopically (data not shown), and minimal (in animal nos. 31 and 58) to mild (in animal no. 54) degree of lymphocytolysis in cortex was observed, which correlated histologically. The gross lesions observed in thymus was an incidental finding and one that should not be considered treatment related since no dose dependent trend was noticed.

##### 3.5.4.3 Histopathology

**FIGURE 3:**
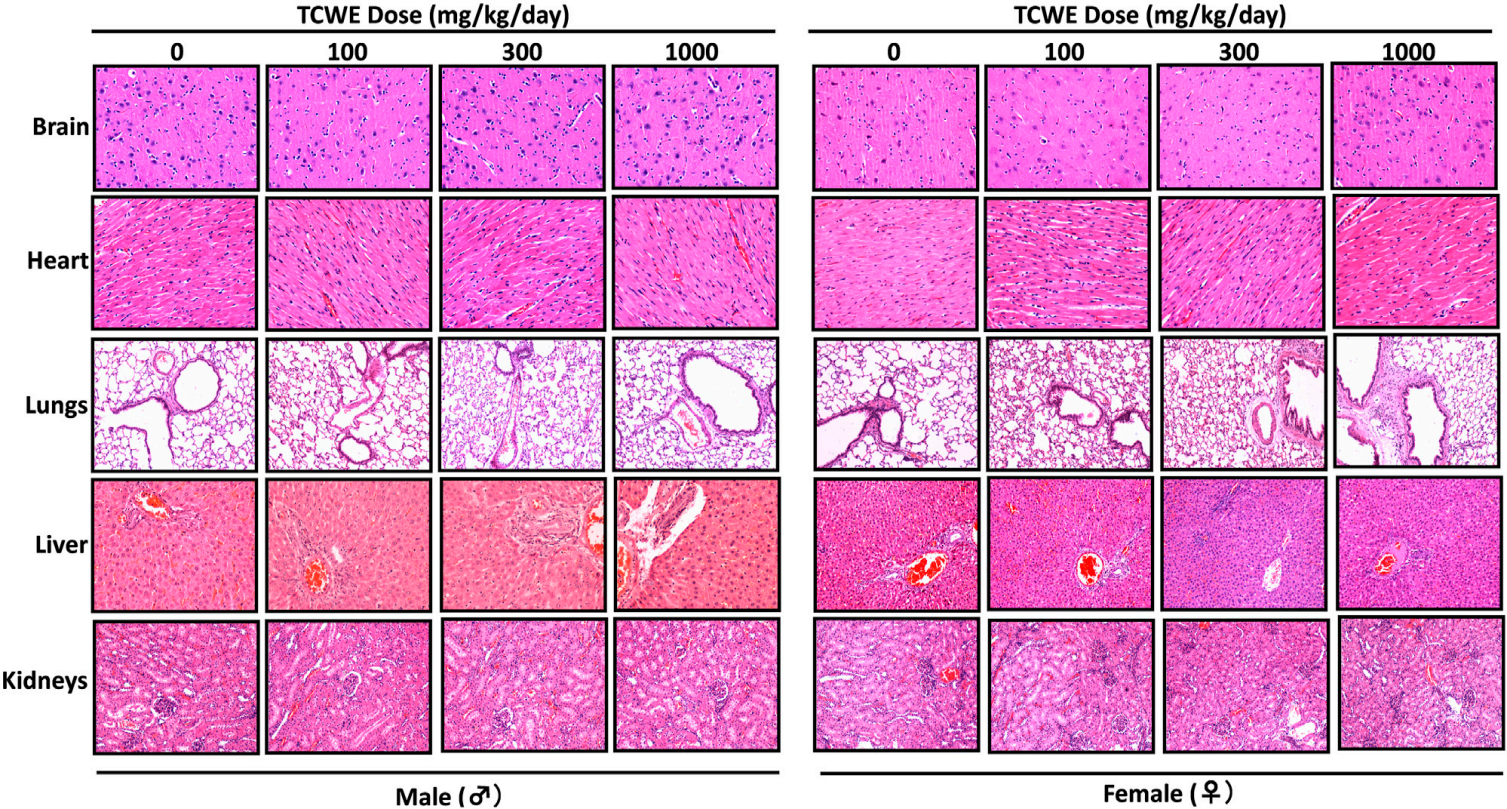
Histopathological effect of TCWE dosing on vital organs Representative micrographs from histopathological analysis of the vital organs, namely, brain, heart, lungs, liver and kidneys from male and female study animals receiving different daily doses (100, 300 and 1000 mg/kg/day) of TCWE for 28 days.

**FIGURE 4:**
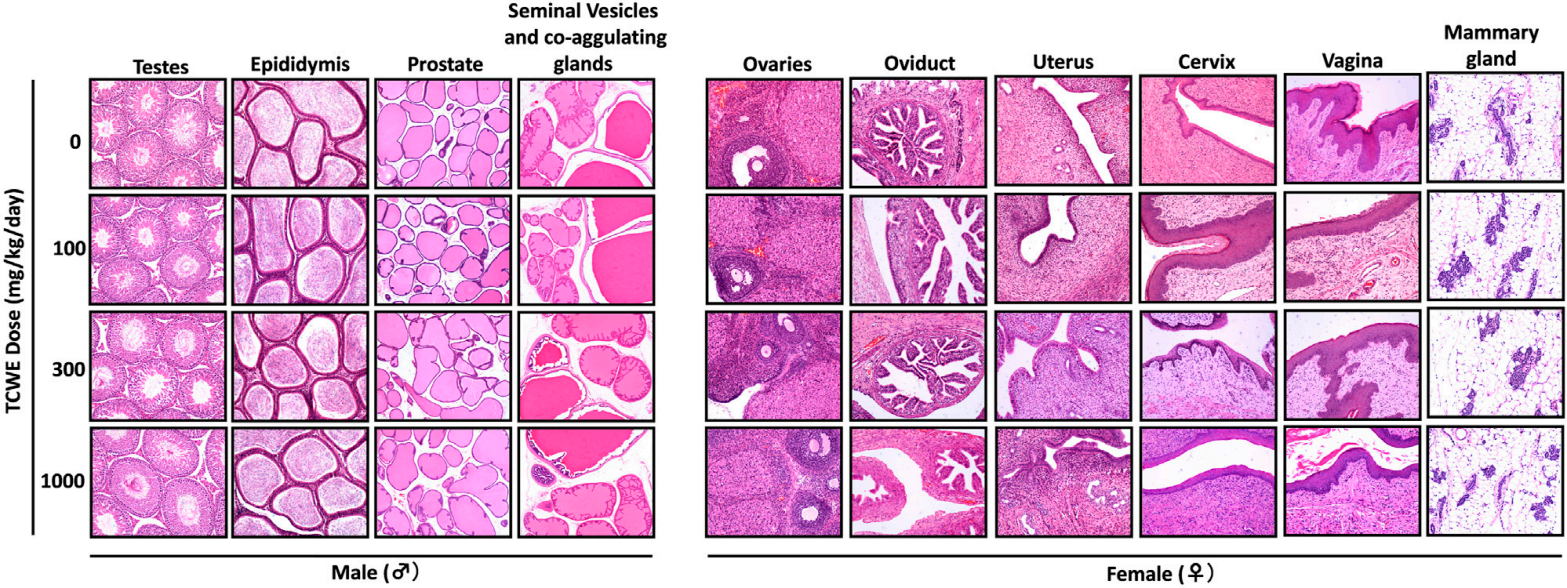
Histopathology of reproductive organs from animals receiving repeated TCWE dosing Representative histopathological micrographs of different parts of the male (testes, epididymis, prostrate and seminal vesicles) and female (ovaries, oviduct, uterus, cervix, vagina and mammary gland) reproductive systems from the study animals receiving different daily doses (100, 300 and 1000 mg/kg/day) of TCWE for 28 days.

**FIGURE 5:**
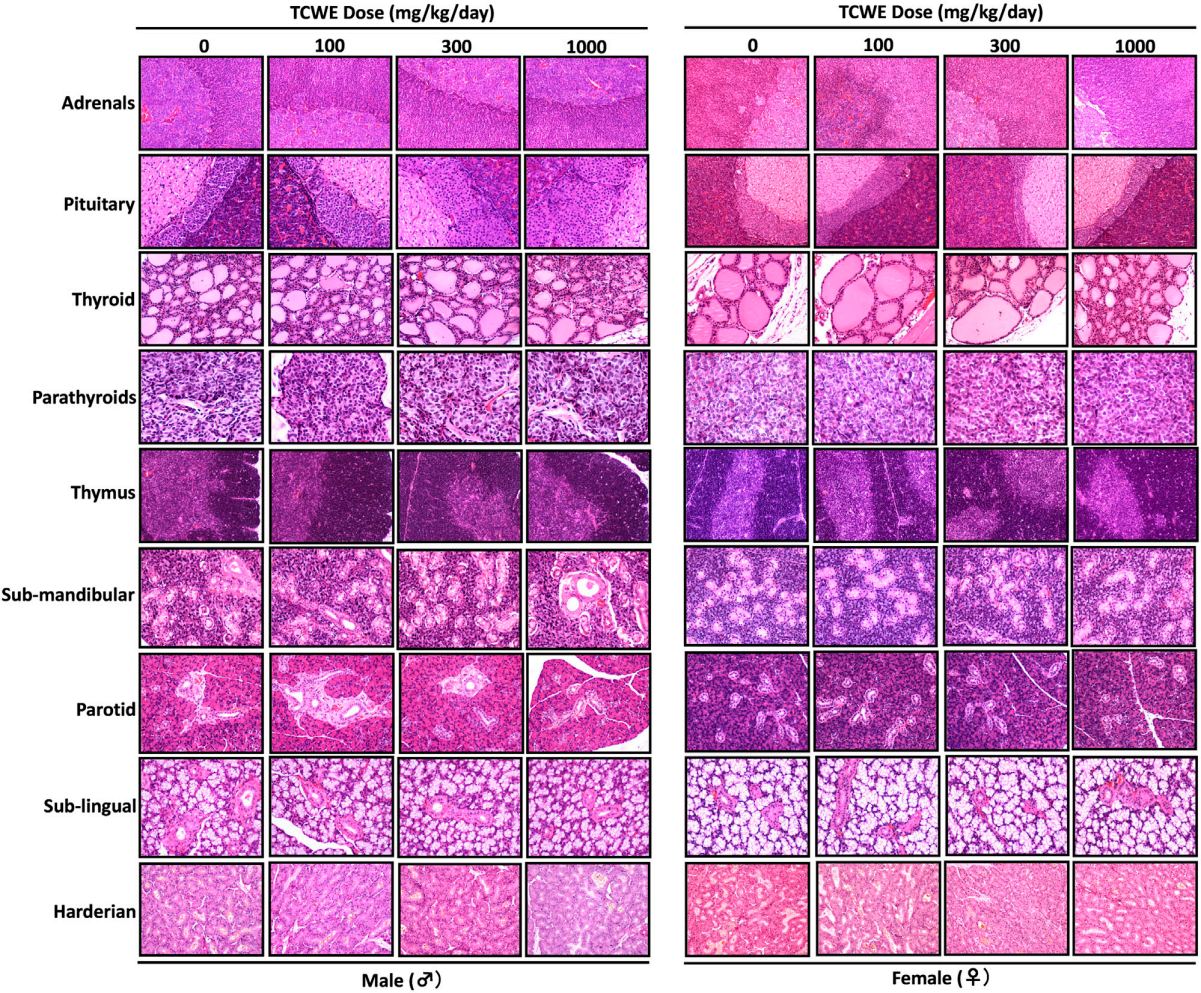
Effect of repeated TCWE dosing on the histopathology of different exocrine and endocrine glands Representative micrographs of histopathology of various exocrine (sub-mandibular, parotid, sub-lingual and harderian) and endocrine (adrenal, pituitary, thyroid, parathyroid and thymus) glands from male and female study animals receiving 100, 300 and 1000 mg/kg/day of TCWE for 28 days.

**FIGURE 6:**
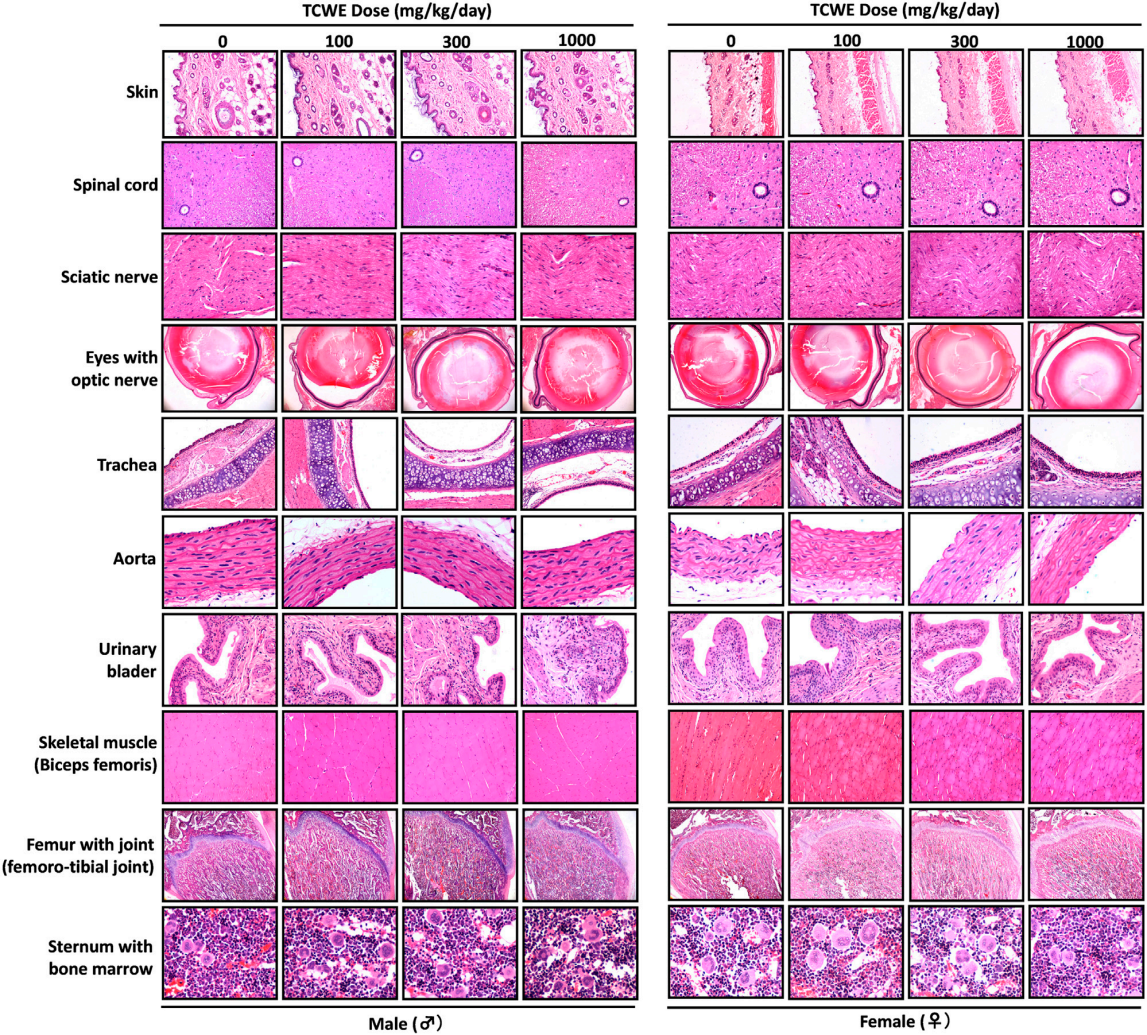
Histopathological analysis of repeated TCWE dosing induced effects in different organs Effect of repeated TCWE dosing on different organs, like, skin, spinal cord, sciatic nerve, eyes with optic nerve, trachea, aorta, urinary bladder, skeletal muscle, femur with joint and sternum with bone marrow shown through representative histopathological micrographs of male and female study animals receiving 100, 300 and 1000 mg/kg/day of the test article for 28 days.

**FIGURE 7:**
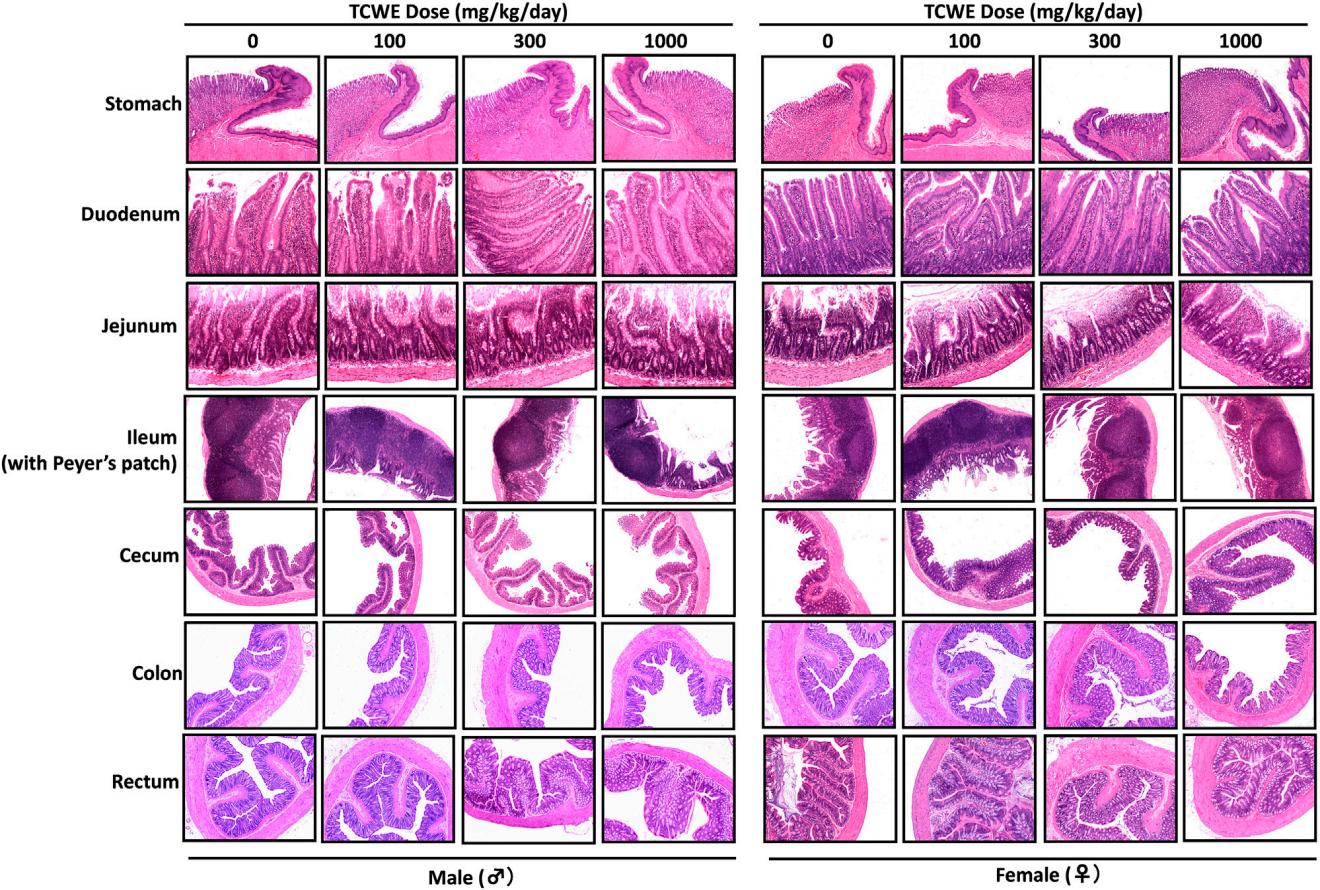
Histopathology of repeated TCWE dosing in different organs of alimentary canal Histopathological effect of repeated TCWE doses on different parts of alimentary canal, like, stomach, duodenum, jejunum, ileum (with Peyer’s patch), cecum, colon and rectum of male and female study animals receiving 100, 300 and 1000 mg/kg/day of the test article for 28 days, shown through representative micrographs.

Different organs from the terminally sacrificed study animals of either sex from the main (G1—G4) groups were subjected to histopathological analysis. Such investigation on the organs of animals belonging to the recovery groups (G5-R and G6-R) was conducted only when required based on the gross pathological observation, as in case of thymus, mentioned above (Figure 1A). Oral administration of TCWE to the study animals of both sexes, for 28 consecutive days did not elicit any treatment-related local or systemic (gross or histopathological) adverse effects on any organs/tissues up to the highest dose (1000 mg/kg/day) (Figure 3; Figure 4; Figure 5; Figure 6; Figure 7).

Some random histopathological aberrations, such as, alveolar osseous metaplasia, MNC infiltration in liver, kidney, interstitial of prostrate and epididymis, lymphocytolysis in the cortex of the thymus and mesenteric lymph node, presence of basophilic and mineralized tubules in the kidney cortex, excess cortical tissue in adrenals, and keratinized cyst in the non-glandular squamous tissue of the stomach, were observed in some of the study animals of either sex in the control and TCWE high dose groups. These histopathological findings did not exhibit any dose dependent co-relation and therefore, considered as spontaneous/incidental and/or congenital in nature unrelated to TCWE administration. Moreover, such histopathological peculiarities have been widely reported as background pathology of laboratory rats in literature.

Thus, gross and histopathological evaluations of organs/tissues from the study animals did not reveal any histopathological findings indicative of treatment-related effect. Therefore, under the conditions of the study, it is concluded that No-Observed-Adverse-Effect-Level (NOAEL) for the test item TCWE, administered to the study animals, Sprague-Dawley rats through oral gavage route for 28 consecutive days could be more than the high dose (1000 mg/kg/day) used in the present study.

## 4 Discussion

*T. cordifolia* is a well-known medicinal plant widely used in Ayurveda, the traditional Indian system of medicine for treating fever, skin diseases, urinary problems, dysentery, diabetes, etc., among several other ailments. This plant is rich in different classes of phytochemicals, like, alkaloids, terpenoids, lignans and steroids that confer its pharmacological efficacies against oxidative stress, microbial infections, diabetes, hepatic disorders, hyperlipidemia, wound healing and several other ailments (Sharma et al., 2019). However, few recent studies have reported apparent hepatotoxicity in clinical subjects who consumed *T. cordifolia* (Nagral et al., 2021; Kulkarni et al., 2022). These reports associating hepatotoxicity with *T. cordifolia* have been rebutted through critical discussions in letters to the editors (Balkrishna et al., 2022a; Bakrishna et al., 2022). Nevertheless, scientifically it is of utmost pertinence that non-clinical safety of *T. cordifolia* is evaluated following good laboratory practice (GLP) as per OECD guidelines for toxicological validation of drugs, much like what has been done for *Withania somnifera* (Balkrishna et al., 2022b). The current study was, thus, conducted in this context in compliance with OECD 407 guidelines, that mandates the evaluation of toxicity resulting from repetitive dosage and subsequent recovery. Accordingly, a total of 72 Sprague-Dawley rats (36 males and 36 females) were randomly allocated to six different dose groups (4 main and 2 recovery groups). Each main group (G1, G2, G3 and G4) and recovery group (G5-R and G6-R) consisted of 6 animals/sex. The animals allocated to group G1/G5 received vehicle control whereas those assigned to groups G2, G3 and G4/G6 received 100, 300 and 1000 mg/kg/day of TCWE, respectively, through oral gavage consecutively for 28 days. Subsequently, study animals in groups G5-R and G6-R were allowed a 14-day treatment-free recovery. No mortality or treatment related clinical signs were noticed, such as changes in body weight, food consumption, ophthalmoscopic, neurological and behavioral symptoms, or any other physiological manifestations, routinely determined through hematological, blood biochemical and urine analyses. Importantly, no treatment related gross and histopathological observations were noted, thus, establishing the No-Observed-Adverse-Effect-Level (NOAEL) of TCWE to be 1000 mg/kg body weight (high dose) when administered orally to male and female Sprague-Dawley rats for 28 consecutive days followed by a 14-day recovery period.

The physical, chemical and safety characteristics of synthetic medicines can be clearly defined because they usually contain a single active pharmaceutical ingredient (API) (Derkach and Tarasenko, 2021). Hence, their characterizations are fairly simple and straightforward. However, same cannot not be claimed to be true for herbal medicines (Shulammithi et al., 2016; Ghosh, 2018), which are inherently complex due to variability in the plant raw materials (Booker et al., 2018; Scotti et al., 2019). The environmental factors, such as, climate and geophysical conditions directly influence phytoconstitutional (or API content) and inorganic element content profiles of the plants (Sarrou et al., 2018; Kohzadi et al., 2019). This, eventually manifests as variations in the efficacy and safety profiles of the herbal preparations (Derkach and Tarasenko, 2021). Therefore, it is all the more important to establish a seamless traceability between the efficacy and safety of any herbal preparation, and phytoconstitutional fingerprinting is the best way to establish such a reference signature. Compositional analysis of the aqueous extract of *T. cordifolia* stem, TCWE, used in this study showed that it contains magnoflorine and β-ecdysone. The quaternary aporphine alkaloid, magnoflorine, is known for its multiple pharmacological activities, such as, anti-diabetic, antioxidant, antifungal and hypotensive, to name a few (Xu et al., 2020). Magnoflorine is a potent anti-inflammatory compound with immunomodulatory effects (Sharma et al., 2012) and is also known to improve the phagocytic efficiency of macrophages (Ahmad et al., 2016; 2018). Although, magnoflorine is known to be cytologically safe, its pre-clinical safety evaluation still awaits (Xu et al., 2020). The current study, in a way, establishes the pre-clinical safety of magnoflorine. β-ecdysone is attributed to the anti-osteoporotic activity of *T. cordifolia* and is believed to be of therapeutic importance against osteoporosis and osteoarthritis (Kapur et al., 2010). The β-ecdysone, found in *T. cordifolia*, besides inducing osteogenic transformation (Abiramasundari et al., 2018), can enhance the population of antibody producing B cells in mice spleen (Lafont and Dinan, 2003). The ability of β-ecdysone to affect Renin-Angiotensin system could be involved in attenuating ACE2 receptor mediated SARS-CoV-2 infection (Lafont et al., 2022). Giloy Ghanvati, prepared from hydromethanolic extract of *T. cordifolia*, besides, magnoflorine and β-ecdysone, also contained cordifolioside A and palmatine (Balkrishna et al., 2021a). The phenylpropene disaccharide, cordifolioside A has demonstrated immunostimulatory effect in mice (Maurya et al., 1996), besides, being known for its macrophage stimulation property through increased NADH-oxidase, NADPH-oxidase and myeloperoxidase activities (More and Pai, 2012). The isoquinoline alkaloid, palmatine, present in the medicinal plants from Berberidaceae, Papaveraceae, Ranunculaceae, and Menispermaceae families, is believed to be the active ingredient against inflammation, hypertension, liver-related diseases, jaundice and dysentery (Tarabasz and Kukula-Koch, 2020). Clerodane furano diterpene glycoside from *T. cordifolia* is also reported to be effective as an autophagic and apoptotic agent in colon cancer cell line HCT 116 (Sharma et al., 2019). In the current study, since, HPLC with photo-diode array detector was used, therefore, clerodane diterpenes could not be detected. Nevertheless, the phytoconstitutional validation of TCWE was done through the detection and quantification of magnoflorine and β-ecdysone. A recent repeated dose toxicological study on polysaccharide-rich *T. cordifolia* extract in balb/c mice has demonstrated a safe profile up to 500 mg/kg body weight (Premkumar et al., 2021). In fact, aqueous extract of *T. cordifolia* was found to be have protective effects against alcoholism-induced liver and gastrointestinal toxicities (Sharma and Dabur, 2015). A toxicokinetic profiling would have been an excellent idea, but its execution had serious technical constraints. Single phytocompound present in the extract is very low and upon absorption their amounts reduced further and reached below detectable levels. Hence all attempts to measure the phytoconstituents present in the extract were unsuccessful in detecting them in the first place. Evaluation of chromosome breaking or clastogenic activity is required for determining genomic safety of a new drug. However, genotoxicity is not in the scope of OECD-407 guideline, which was followed to conduct this 28-day repeat dose non-clinical toxicity study. Therefore, evaluation of genotoxicity was not conducted in this study. As per the guidelines of Ministry of AYUSH, Government of India, medicinal plants, mentioned in the classical texts of traditional Indian systems of medicine, need not be subjected to any regulatory toxicological study. The reason we conducted this study as per OECD 407 guidelines is to demonstrate that traditional Indian medicines can also be subjected to similar modes of validation as modern medicines. Moreover, the fact that *T. cordifolia* has been recommended in several Ayurvedic (traditional Indian) medicines for centuries according to classical medicinal texts testifies that this therapeutic plant is most likely be free of deleterious effects, such as, genotoxicity, if used as recommended. In fact, an earlier report showed that the aqueous extract of *T. cordifolia* did not have any clastogenic and DNA damage effects respectively, on the mice bone marrow erythrocytes and peripheral blood lymphocytes (Chandrasekaran et al., 2009). The study by Chandrasekaran et al. has served as a background information for us. However, a battery of genotoxicity assessments, such as, Ames test, *in-vitro* micronucleus assay and hypoxanthine phosphorybosyl transferase assay, would provide better insight into the effect of TCWE on the genome. Therefore, these assessments are included as a part of our next study. Likewise, safety pharmacology, being out of scope of OECD-407 scope, was also not part of this study, although, it has now been included in our upcoming study on TCWE. Thus, taken together, it can be concluded that *T. cordifolia* has a safe sub-acute toxicity profile that warrants for further studies on its sub-chronic and chronic toxicity profiles. In compliance with OECD 407 guidelines, this toxicological study has stringently abided by the regulatory directives, which has left room and at the same time paved the path for other types regulatory studies, such as 28 days repeat toxicological studies in higher models, such as, dogs and even non-human primates. Safety pharmacology and genotoxicity for TCWE *per se* should be conducted for a better understanding of its therapeutic reliability.

## 5 Conclusion

The OECD 407 guideline compliant 28-days repeated dose with 14-day recovery toxicity profiling of the aqueous extract of *Tinospora cordifolia* (Wild.) Hook.f. & Thomson stem showed no treatment related mortality, clinical signs, gross and histopathological aberrations at a high oral dose of 1000 mg/kg/day. Therefore, the No-Observed-Adverse-Effect-Level (NOAEL) under the given experimental conditions was determined to be 1000 mg/kg body weight for both male and female Sprague-Dawley rats. In conclusion, the outcomes of this toxicological study establish the non-clinical safety profile of *T. cordifolia* water extract and also encourage other types of regulatory non-clinical and clinical safety profiling for this important traditional medicinal herb.

## Data Availability

The original contributions presented in the study are included in the article/Supplementary Material, further inquiries can be directed to the corresponding author.
